# Identification of methylation-driven genes prognosis signature and immune microenvironment in uterus corpus endometrial cancer

**DOI:** 10.1186/s12935-021-02038-z

**Published:** 2021-07-10

**Authors:** JinHui Liu, ChengJian Ji, Yichun Wang, Cheng Zhang, HongJun Zhu

**Affiliations:** 1 Department of Gynecology, The First Affiliated Hospital of Nanjing Medical University, Nanjing, 210029 Jiangsu China; 2 Department of Urology, The First Affiliated Hospital of Nanjing Medical University, Nanjing, 210029 Jiangsu China; 3 Women & Children Central Laboratory, The First Affiliated Hospital of Nanjing Medical University, Nanjing, 210029 Jiangsu China; 4 Department of Oncology, The Third People’s Hospital of Nantong, Nantong, 226001 Jiangsu China

**Keywords:** TCGA-UCEC, DMDGs, Prognosis, Tumor microenvironment, Immunotherapy

## Abstract

**Background:**

Uterus corpus endometrial cancer (UCEC) is the main malignant tumor in gynecology, with a high degree of heterogeneity, especially in terms of prognosis and immunotherapy efficacy. DNA methylation is one of the most important epigenetic modifications. Studying DNA methylation can help predict the prognosis of cancer patients and provide help for clinical treatment. Our research aims to discover whether abnormal DNA methylation can predict the prognosis of UCEC and reflect the patient's tumor immune microenvironment.

**Patients and methods:**

The clinical data, DNA methylation data, gene expression data and somatic mutation data of UCEC patients were all downloaded from the TCGA database. The MethylMix algorithm was used to integrate DNA methylation data and mRNA expression data. Univariate Cox regression analysis, Multivariate Cox regression analysis, and Lasso Cox regression analysis were used to determine prognostic DNA methylation-driven genes and to construct an independent prognostic index (MDS). ROC curve analysis and Kaplan–Meier survival curve analysis were used to evaluate the predictive ability of MDS. GSEA analysis was used to explore possible mechanisms that contribute to the heterogeneity of the prognosis of UCEC patients.

**Results:**

3 differential methylation-driven genes (DMDGs) (PARVG, SYNE4 and CDO1) were considered as predictors of poor prognosis in UCEC. An independent prognostic index was finally established based on 3 DMDGs. From the results of ROC curve analysis and survival curve analysis, MDS showed excellent prognostic ability in TCGA-UCEC. A new nomogram based on MDS and other prognostic clinical indicators has also been successfully established. The C-index of the nomogram for OS prediction was 0.764 (95% CI = 0.702–0.826). GSEA analysis suggests that there were differences in immune-related pathways among patients with different prognosis. The abundance of M2 macrophages and M0 macrophages were significantly enhanced in the high-risk group while T cells CD8, Eosinophils and Neutrophils were markedly elevated in the low-risk group. Meanwhile, patients in the low-risk group had higher levels of immunosuppressant expression, higher tumor mutational burden and immunophenoscore (IPS) scores. Joint survival analysis revealed that 7 methylation-driven genes could be independent prognostic factors for overall survival for UCEC.

**Conclusion:**

We have successfully established a risk model based on 3 DMDGs, which could accurately predict the prognosis of patients with UCEC and reflect the tumor immune microenvironment.

**Supplementary Information:**

The online version contains supplementary material available at 10.1186/s12935-021-02038-z.

## Introduction

Uterus corpus endometrial cancer (UCEC), as the sixth most common malignant tumor in women worldwide, has an increasing morbidity and mortality year by year [1–3]. At present, surgery combined with adjuvant therapy (radiotherapy and/or chemotherapy) is the standard treatment for UCEC patients [4, 5]. As an emerging adjuvant treatment, immunotherapy brings hope to patients who cannot choose standard treatment methods [6, 7]. Nevertheless, the prognosis of UCEC patients is still very different. Some patients with recurrent or advanced tumors (stage III and IV) still have a poor prognosis [8].

Clinical staging is now the most mainstream factor predicting the prognosis of UCEC patients. However, it can often be found in the clinic that patients with the same stage have completely different survival outcomes [9, 10]. Therefore, finding reliable molecular markers is an urgent need for personalized treatment and improving clinical efficacy.

In addition, in terms of treatment options, for patients who suffered from tumor progression after standard treatment, targeted therapy and immunotherapy may be a promising rescue method. However, only a small percentage of patients can really benefit from immunotherapy. Therefore, it is also very urgent to find biomarkers that can predict the response of patients to immunotherapy.

DNA methylation is an important epigenetic modification catalyzed by DNA methyltransferases which add a methyl group to position 5 of the cytosines present in the transcription regulatory regions of genomic DNA using *S*-adenosyl methionine as donor molecule. Generally, hypermethylation correlates with gene silencing while hypomethylation means gene activation. Hypermethylation in promoter regions of some important genes, such as tumor suppressor genes and DNA repair genes, leading to down-regulation of their expression, may result in abnormal cell differentiation and regulation and DNA damage that cannot be repaired. Which is believed to be closely related to the occurrence and development of cancer [11]. With the deepening of research, abnormal changes in DNA methylation are considered potential biomarker for cancer risk assessment, early diagnosis and prognostic monitoring. As early as 2016, Balázs et al. had confirmed that abnormal DNA methylation was closely related to the prognosis of ER+/HER2− breast cancer [12]. Subsequently, abnormal DNA methylation was found to be an independent prognostic biomarker for a variety of tumors (including esophageal cancer, lung cancer and head and neck squamous cell carcinoma) [13–15]. In UCEC, many studies have shown that the occurrence and development of tumors are closely related to abnormal DNA methylation. The genome-wide DNA methylation profile of endometrial cancer clarifies the importance of DNA methylation changes in the process of endometrial cancer genesis [16]. In 2018, abnormal methylation levels of KLF4 and HS3ST2 genes were revealed to be important in the development of endometrial hyperplasia and UCEC [17]. The abnormal methylation of PTEN, APC, CDO1, SOX11 was found to be an independent risk factor for UCEC [18–20].

In recent years, with the continuous advancement of high-throughput sequencing technology, the combined analysis of multiple omics has become more and more widely used in tumor research. As a new tumor marker with stability, reversibility and high frequency, abnormal DNA methylation changes have been used by many researchers to combine gene expression profiles and clinical data to develop prognostic tumors model with more accurate prediction capabilities. By integrating methylation and mRNA expression profile data, some molecular prognostic models with high prognostic ability have been established and verified in gastric cancer and colon cancer [21–23]. However, no one has made similar research in UCEC.

In addition, studies have shown that DNA methylation profiles can reflect the immune microenvironment of tumors, which is closely related to the efficacy of immunotherapy [24]. Therefore, the purpose of this article is to use the methylation data, gene expression data and clinical data from The Cancer Genome Atlas (TCGA) Data Portal to construct a molecular model that can effectively predict the prognosis of UCEC patients and reflect the tumor immune microenvironment.

## Material and methods

### Ethics committee approval

All data were downloaded from the public databases hence it was not required to obtain additional ethical approval for our study.

### Data acquisition and processing

Three types of TCGA-UCEC data (including patient clinical information, methylation data based on Illumina Infinium HumanMethylation 450 platform and level-3 mRNA expression files were obtained from TCGA (https://tcga-data.nci.nih.gov/tcga/; accessed August 2020). Then, we retained patients with complete clinical data including age, stage, grade and histological type, methylation data or mRNA expression data for further study.

We obtained the complete clinical data of 544 patients from the TCGA database. The clinicopathological characteristics of the patients included in the study are shown in Additional file 12: Table S1. At the same time, we obtained DNA methylation data of 439 tumor tissues and 46 adjacent normal tissues and mRNA expression data of 552 tumor tissues and 23 adjacent normal tissues from the TCGA database. In the end, we retained 429 patients with complete survival data, methylation data and mRNA expression data for further research.Text: “Additional files: As per journal instructions, we have ignored to process the Original data files "different expression gene data.xls"; "Supplymentary data.xls"; "Gene expression data.txt" and "methylation data.txt" given in the submission package. Kindly check and confirm.we have checked it.Thanks.

### Identification of differential methylation-driven genes (DMDGs)

By using the “Limma” package in the R statistics software, we obtained the difference in mRNA expression between tumor samples and normal samples. mRNAs satisfying both |fold change (FC)| > 1 and adjusted p < 0.05 were defined as significantly differentially expressed. The MethylMix algorithm was used to integrate the DNA methylation data and differentially expressed mRNA data into the same sample for correlation analysis. Differential methylation-driven genes were finally identified by using the “MethylMix” package.

### Functional enrichment analysis

By using the ‘clusterProfiler’ package in the R statistics software, we performed gene ontology (GO) enrichment analysis and Kyoto Encyclopedia of Genes and Genomes (KEGG) pathways enrichment analysis on the obtained DMDGs. We set an adjusted p < 0.05 as the cut-off value to screen for meaningful functional events.

### Establishment of a methylation-driven signature (MDS) based on DMDGs

After matching the complete survival data with methylation and mRNA expression data, we acquired 429 patients. Then we acquired data from 429 patients (entire cohort) randomly assigned 216 patients as the training cohort with the help of “caret” R package. The remaining 213 patients were assigned as the testing cohort. Then, in the training cohort, the univariate Cox regression analysis and the multivariate Cox regression analysis were used to identify genes with prognostic value from differentially expressed DMDGs. We used the “survival” package in R software for Cox regression analysis. Next, “glmnt” package was used to perform the Lasso Cox regression to avoid overfitting.

We then utilized multivariate Cox model to verify the correlation between gene expression of patient’s survival status. Besides, multivariate Cox model can also assign the weight of each involved gene in reflect the prognosis information of patients. We constructed a risk methylation-driven signature (MDS) based on the correlation coefficient of the key genes from the multivariate Cox regression. The formula of MDS was defined as follows: β_1_ × gene_1_ expression + β_2_ × gene_2_ expression + $\cdots$ + β_n_ × gene_n_ expression, where β corresponded to the correlation coefficient. According to our prognostic model, each patient would get a risk score. We set the median risk score as the cutoff value for dividing UCEC patients into a high-risk group and a low-risk group.

### Evaluation of the MDS

Firstly, we conducted overall survival (OS) curves and time-dependent receiver operating characteristic (ROC) curve analysis in the training cohort. OS curves were plotted by the Kaplan–Meier (K–M) method. ROC curve analysis was obtained through the “survivalROC” package. Next, we used the same methods to evaluate the prognostic value of MDS in the testing cohort. In order to more comprehensively evaluate the application ability of the prognostic MDS we constructed in UCEC patients, we have drawn the OS curves of the high-risk group and the low-risk group of patients with different clinical characteristics in the entire cohort.

To further evaluate whether our model could be used as an independent prognostic factor, we included age, stage, histological type, grade and MDS as independent variables. And then univariate Cox regression analysis and multivariate Cox regression analysis were carried out to identify the prognostic performance of these factors in the entire cohort. We also made multivariate ROC curves at different observation times to visualize the diagnostic performance of each factor. Then, the correlation between the prognostic model (MDS) and each clinical feature was analyzed to illustrate the reliability of the model we built in the entire cohort. Then, we explored the phenotypic differences between patients in the high-risk group and patients in the low-risk group in the entire cohort. Which includes the expression of risk genes, the methylation level of risk genes, the level of mRNAsi, Tumor mutational burden (TMB), the expression of m6A genes, immunophenoscore (IPS) and the infiltration of immune cells in the entire cohort. Among them, the results of mRNAsi in TCGA-UCEC were obtained from a previous study [25]. In order to obtain more reliable somatic mutation results, “VarScan2” software was used to identify tumor cell mutations. The “maftools” package was used to read and visually analyze the somatic variants of each sample. TMB was defined as the number of somatic variants per megabase of genome. The infiltration of immune cells was obtained through the CIBERSORT tool [26]. Scores of immune and stromal cells were also calculated by Estimation of Stromal and Immune cells in Malignant Tumours using Expression data (ESTIMATE) algorithm. What’more, IPS, which could predict the efficacy of immunotherapy, was calculated as described in the previous research [27].

### Quantitative real-time RT-PCR (qRT-PCR) analysis

qRT-PCR was used to further verify the expression of prognostic genes in tissue samples. After obtaining the informed consent of each patient and the approval of the Institutional Review Committee of Nanjing Medical University, we collected 13 pairs of normal tissues and cancer tissues. These tissues were stored at − 80 °C before using.

Total RNA was extracted by using TRIzol reagent (Thermo Fisher Scientific, Waltham, MA, USA). And the NanoDrop 2000 Spectrophotometer (Thermo Scientific, Wilmington, DE, USA) was used to measure the concentration of isolated RNA. High-capacity cDNA reverse transcription kits (Thermo Fisher Scientific) were used for reverse transcription of RNA extracted. The program of qRT-PCR was performed with the help of the SYBR Green PCR kit (Thermo Fisher Scientific) on Light Cycler 480 (Roche, Switzerland). The 2^–ΔΔCt^ method was used to assess the relative expression level of mRNAs. The primer sequence information is in Additional file 13: Table S2.Additional file: As per journal requirements, every additional file must have a corresponding caption. In this regard, please be informed that the caption of Additional file 13 was taken from the additional e-file itself. Please advise if action taken appropriate and amend if necessary.we have checked it.Thanks.

### Construction of prognostic nomogram

After obtaining the results of Cox regression analysis in the independent evaluation of prognostic factors, we used the independent prognostic factors as parameters for constructing a nomogram with more accurate prognostic performance. The nomogram was carried out by means of “rms” package. We also made calibration curves and calculated C-index to evaluate the accuracy of the nomogram.

### Gene-set enrichment analysis

The genome wide expression profiles of the UCEC in the high risk group and low risk group were employed to identify the biological functions and pathways by Gene-set enrichment analysis (GSEA) with GSEA tool (version 4.0.1) (http://software.broadinstitute.org/gsea/index.jsp) and annotated gene sets (h.all.v7.4.symbols.gmt) as previous research did [28]. The gene sets were filtered using the maximum and minimum gene set size of 500 and 15 genes, respectively. The enriched gene sets were obtained based on Nom.p < 0.05 and FDR < 5% after performing 1000 permutations.

### Joint survival analysis of methylation and gene expression

By means of cox regression analysis and K–M methods, joint survival analysis based on gene expression levels and methylation was carried out.

### Statistical analysis

Statistical analyses of all data utilized in this article were completed by R software (version 3.6.1, https://www.r-project.org/). When the difference met a joint satisfaction of adjust p < 0.05 and |fold changes (FC)| > 1, it was regarded to be statistically significant. Student’s t test was used for continuous variables, while categorical variables were compared with the chi-square (χ^2^) test. The Wilcoxon rank-sum test was utilized to compare ranked data with two categories. The Kruskal–Wallis test was utilized for comparisons among three or more groups. The univariate Cox regression analysis and multivariate Cox regression analysis were used to evaluate the relationship between MGs expression and survival data to establish a prognostic model. The Pearson Correlation Coefficient was used to investigate the correlation between MDS and other parameters. “rms” package of R software was used to create the nomogram. The receiver operating characteristic curves were created by the “survivalROC” package of R software and AUC values were also calculated by this package. If the AUC > 0.60, we would consider this model to have a certain predictive ability. If the AUC > 0.75, we would consider this prediction model to have excellent predictive value. All statistical tests were two-sided and *p* < 0.05 was considered to be statistically significant.

## Results

### Determination of DMDGs and functional enrichment analysis

**Fig. 1:**
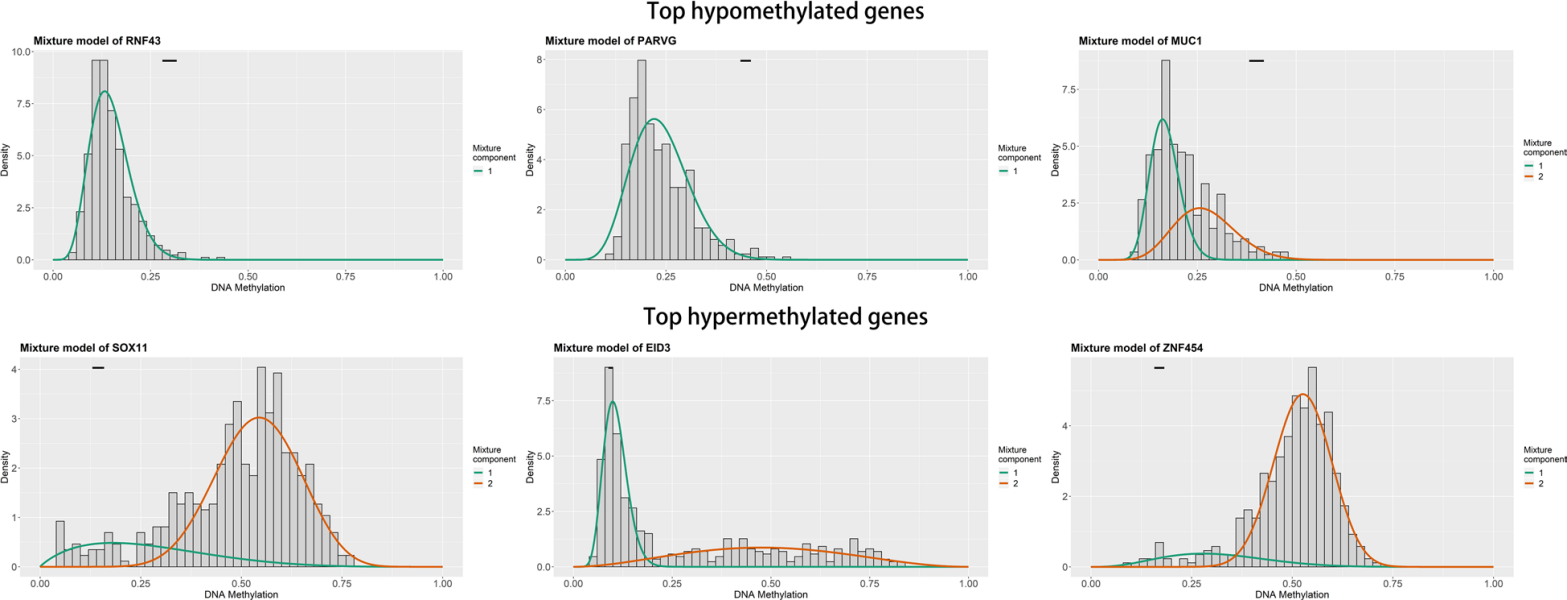
Summary of top methylated genes. The distribution map showing the methylation degree of methylated genes in TCGA-UCEC. The histogram demonstrates the distribution of methylation in the tumor samples (denoted as β-values, where higher β-values represent greater methylation)

**Fig. 2:**
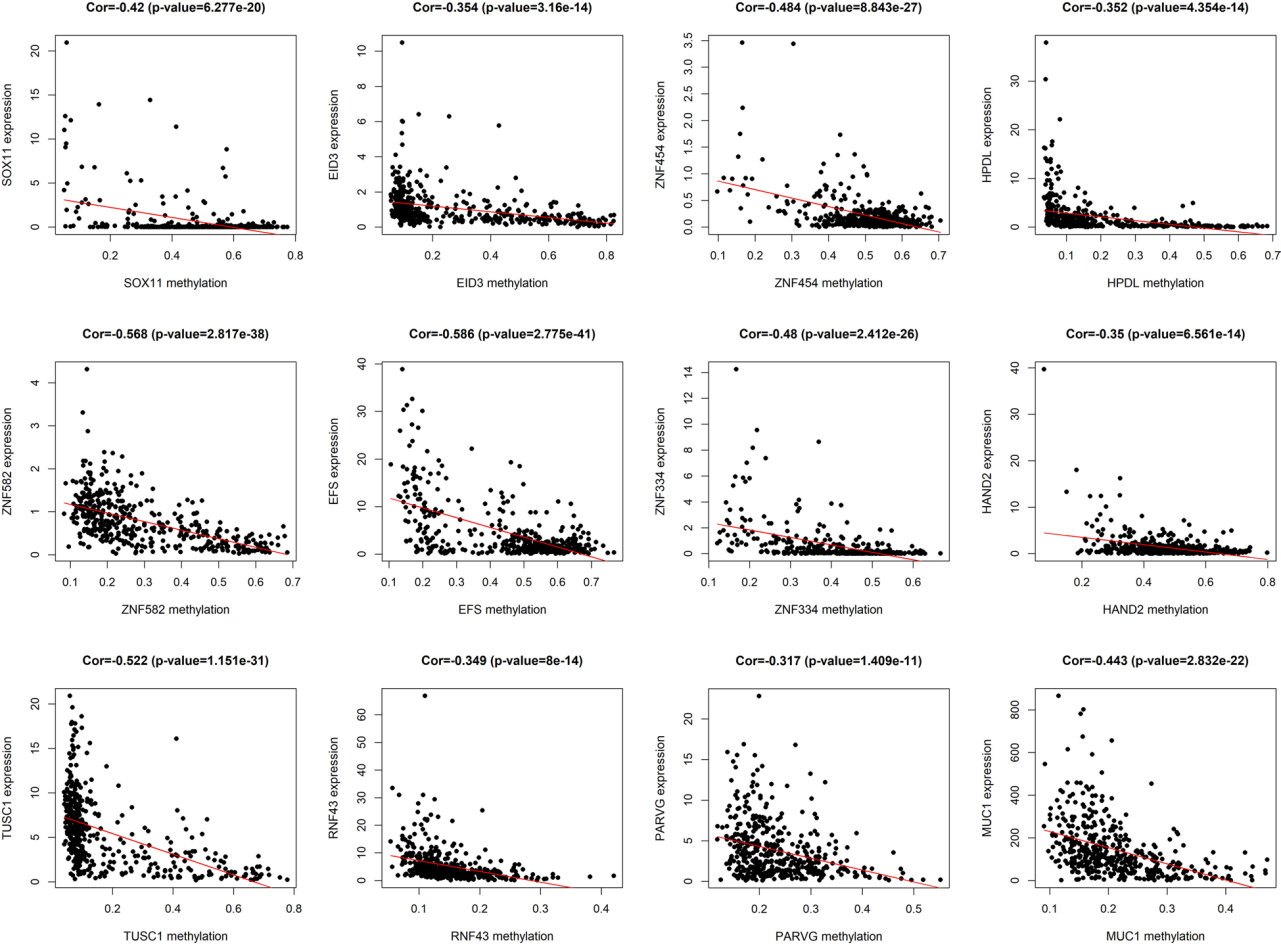
The correlation between gene expression and DNA methylation of top hypermethylated and hypomethylated genes. Average β-values are presented on the x-axis, log2 FPKM gene expression values are presented on y-axis

**Fig. 3:**
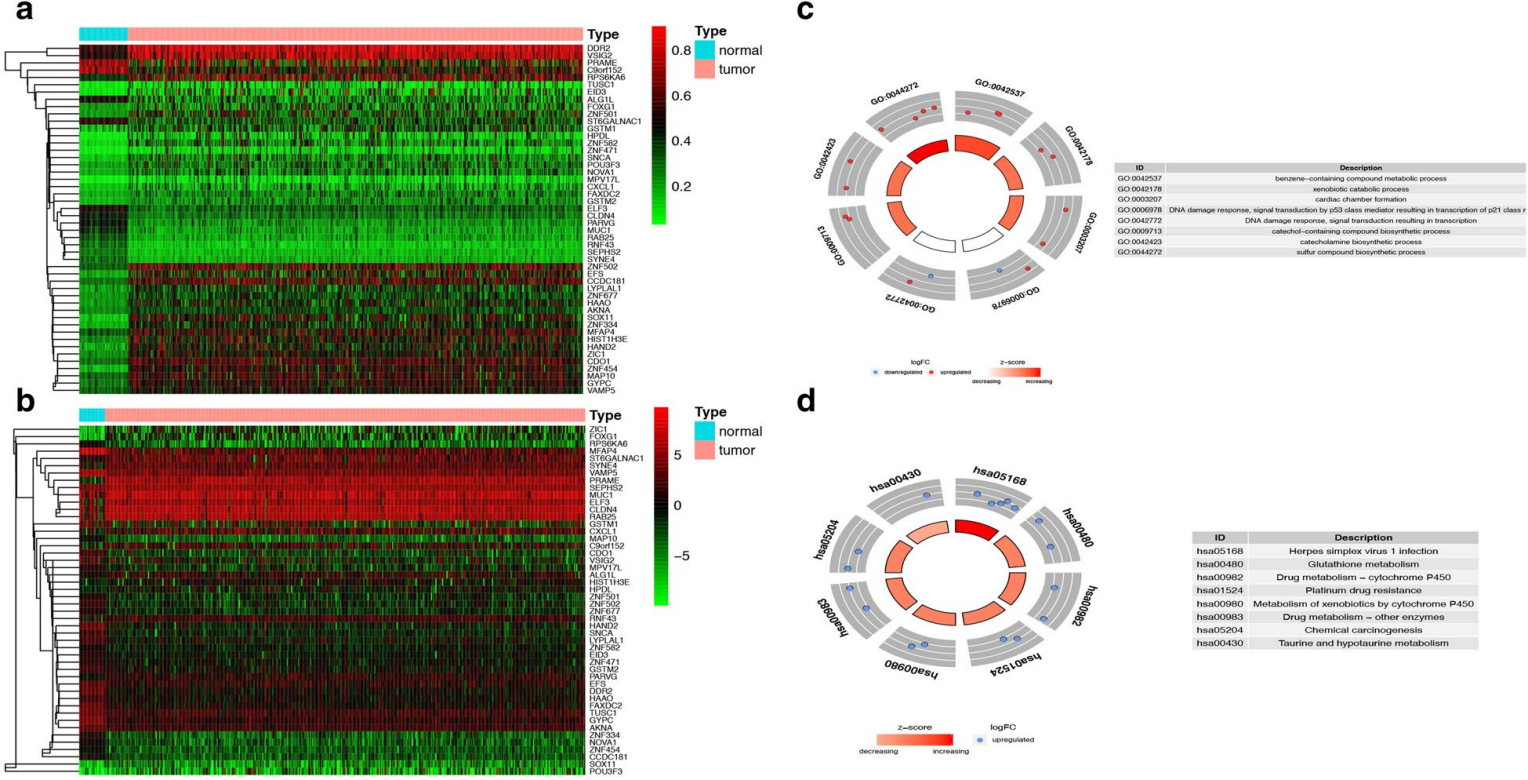
Heatmap and functional enrichment analysis of methylation-driven genes in TCGA-UCEC. **A** Methylation values (β-values). The color change from green to red in the heatmap illustrates the trend from low to high methylation. **B** The expression of the methylation-regulated genes. The color change from green to red in the heatmap illustrates the trend from low to high expression. **C** The outer circle represents the methylation values (logFC) of methylated genes in each enriched GO (gene ontology) term: red dots on each GO term indicate upregulated genes, whereas blue dots indicate downregulated genes. The inner circle indicates the significance of GO terms (log10‐adjusted p values). **D** Kyoto Encyclopedia of Genes and Genomes (KEGG) pathways

The total workflow is shown in the following figure (Additional file 1: Figure S1). A total of 4774 genes were found to be abnormally expressed in tumor tissues, including 2724 up-regulated genes and 2050 down-regulated genes. We used the MethylMix algorithm to integrate the methylation data and gene expression data of each sample. Then, a mixed model including DNA methylation and mRNA expression was established. A Wilcoxon rank test was performed to determine the methylation-driven genes that were significantly differentially expressed. In the end, 48 methylation-driven genes were found to be significantly differentially expressed (|logFC| > 1; p < 0.05; Cor < − 0.3). Among these DMDGs, 12 were hypomethylated genes and the others were hypermethylated genes (Table 1). Figure 1 shows the distribution map of several genes with highest methylation levels. Figure 2 shows the correlation between gene expression and DNA methylation of top hypermethylated and hypomethylated genes. The methylation levels and gene expression levels of DMDGs in each sample we included in the study were displayed in the form of heat maps (Fig. 3A, B).

**Table 1:** Methylation-driven genes in TCGA-UCEC

Gene	normalMean		TumorMean		logFC		pValue		cor			corPavlue
ZIC1	0.24388423		0.480909		0.97956742		3.86E−27		− 0.358903			1.31E−14
DDR2	0.53360917		0.78946164		0.5650857		5.41E−27		− 0.3282384			2.46E−12
ZNF454	0.16962317		0.49986881		1.55921625		5.91E−27		− 0.4837467			8.84E−27
PARVG	0.44800915		0.2361113		− 0.9240611		2.47E−26		− 0.3172147			1.41E−11
SEPHS2	0.28267773		0.16730021		− 0.756719		3.28E−26		− 0.4605881			4.00E−24
GYPC	0.28342815		0.5133146		0.85686022		8.16E−26		− 0.3504962			5.83E−14
HIST1H3E	0.25147232		0.48165462		0.9375994		9.75E−26		− 0.3425503			2.30E−13
HAND2	0.20123389		0.46384627		1.20477346		1.36E−25		− 0.3498187			6.56E−14
CDO1	0.29599759		0.56098126		0.92236715		1.82E−25		− 0.455063			1.61E−23
ELF3	0.50067406		0.32635135		− 0.6174457		2.48E−25		− 0.3996758			4.88E−18
CCDC181	0.28894174		0.55897974		0.95201739		3.26E−25		− 0.3601706			1.04E−14
ZNF582	0.11179343		0.29681801		1.4087432		8.65E−25		− 0.5675056			2.82E−38
CLDN4	0.4338084		0.28653612		− 0.598341		1.64E−24		− 0.3226787			5.99E−12
MUC1	0.39993189		0.21303751		− 0.9086469		4.80E−24		− 0.4433414			2.83E−22
RNF43	0.29871314		0.14721458		− 1.0208401		5.08E−24		− 0.3486804			8.00E−14
SOX11	0.14442811		0.4895337		1.76105668		8.68E−24		− 0.4199562			6.28E−20
ZNF334	0.17865714		0.41878261		1.229008		9.40E−24		− 0.4800659			2.41E−26
VSIG2	0.50192277		0.74041012		0.56085922		1.33E−23		− 0.5896991			6.59E−42
SYNE4	0.30599367		0.18129516		− 0.7551614		4.99E−23		− 0.4613683			3.28E−24
MAP10	0.31229235		0.52780824		0.75711664		1.47E−22		− 0.5832404			8.02E−41
AKNA	0.25605955		0.36468253		0.51016171		4.19E−22		− 0.3612481			8.57E−15
RAB25	0.33919528		0.20764432		− 0.7080017		1.53E−21		− 0.5555928			1.94E−36
VAMP5	0.32375158		0.47780114		0.56152305		2.60E−21		− 0.3432457			2.04E−13
ZNF677	0.2289589		0.37276287		0.70316948		3.67E−20		− 0.5031252			3.66E−29
MFAP4	0.34031638		0.52691515		0.63069407		6.07E−20		− 0.3764796			4.99E−16
HAAO	0.22510224		0.42993225		0.93352892		2.24E−19		− 0.3908538			2.96E−17
EFS	0.19074443		0.45628049		1.25828004		1.24E−18		− 0.5860014			2.78E−41
ZNF502	0.32342026		0.55929393		0.79019662		4.87E−18		− 0.7615427			3.34E−83
ST6GALNAC1	0.52377837		0.35028925		− 0.5804098		1.23E−17		− 0.3588641			1.32E−14
SNCA	0.14417966		0.29249887		1.02056345		2.99E−17		− 0.3170668			1.44E−11
C9orf152	0.72117283		0.50374775		− 0.5176436		5.36E−17		− 0.4220685			3.92E−20
ALG1L	0.48252392		0.29147766		− 0.7272152		1.31E−15		− 0.4049484			1.62E−18
LYPLAL1	0.21600689		0.37575249		0.79870533		1.37E−15		− 0.5716257			6.26E−39
FAXDC2	0.18743077		0.27804419		0.56895638		2.06E−14		− 0.5090998			6.27E−30
GSTM2	0.1676375		0.25583059		0.60984384		1.08E−13		− 0.37316			9.39E−16
RPS6KA6	0.3918945		0.58429377		0.57622858		1.14E−13		− 0.593005			1.79E−42
GSTM1	0.18134566		0.36078245		0.99238695		3.75E−13		− 0.5991552			1.53E−43
ZNF471	0.07742318		0.15964683		1.04404648		4.11E−12		− 0.4331758			3.12E−21
ZNF501	0.21907069		0.39138671		0.83719832		1.18E−11		− 0.5880791			1.24E−41
HPDL	0.07659738		0.21061474		1.45923942		5.54E−11		− 0.3521578			4.35E−14
EID3	0.09448771		0.27900888		1.5621124		1.44E−10		− 0.3539739			3.16E−14
PRAME	0.71350801		0.48887503		− 0.5454639		3.75E−09		− 0.6966287			3.82E−64
POU3F3	0.20637371		0.30865963		0.58075762		2.24E−08		− 0.3212335			7.52E−12
MPV17L	0.06603454		0.13922797		1.07615617		5.38E−08		− 0.3533641			3.52E−14
NOVA1	0.18514835		0.29550643		0.67450785		3.74E−07		− 0.3407751			3.10E−13
TUSC1	0.08324606		0.18763643		1.17248603		0.00048328		− 0.5222237			1.15E−31
FOXG1	0.22293814		0.32155696		0.52843088		0.00791315		− 0.4117017			3.83E−19
CXCL1	0.14772043		0.22793677		0.62576431		0.01593495		− 0.3218651			6.81E−12

In order to explore the possible role of these DMDGs in the occurrence and development of UCEC, GO enrichment analysis and KEGG enrichment analysis of DMDGs were done. From Fig. 3C, we can see that these DMDGs were mainly enriched in eight GO terms, including benzene-containing compound metabolic process, xenobiotic catabolic process, cardiac chamber formation, DNA damage response, signal transduction resulting in transcription, catechol-containing compound biosynthetic process and so on. KEGG enrichment analysis indicated that these DMDGs may be related to Herpes simplex virus 1 infection, Glutathione metabolism, Drug metabolism—cytochrome P450, Platinum drug resistance, Metabolism of xenobiotics by cytochrome P450, Chemical carcinogenesis and so on (Fig. 3D).

### Establishment of an independent prognostic index

Firstly, the patients included in the study were randomly divided into training cohort (n = 216) and testing cohort (n = 213) according to the composition of each clinical feature. Secondly, 3 DMDGs (PARVG, SYNE4 and CDO1) with prognostic significance were identified by univariate Cox regression analysis in the training group. The results of Lasso Cox regression analysis showed that the three prognostic DMDGs (PARVG, SYNE4 and CDO1) had no obvious overfitting (Additional file 2: Figure S2A, B). The relationship between the three DMDGs and the OS of UCEC patients was also confirmed in the multivariate Cox regression analysis. Then, we further verified the expression of these three genes in 13 pairs of normal tissues and tumor tissues. We found that SYNE4 and PARVG were significantly higher expressed in tumor tissues, while CDO1 showed no significant difference (Additional file 2: Figure S2C–E).

According to the 3 DMDGs and their corresponding coefficients in multivariate Cox regression analysis, an independent prognostic index (MDS) was established. The formula of MDS was defined as follows: 3.75767726426642 × PARVG expression + 5.91085180306775 × SYNE4 expression + 5.50757519645672 × CDO1 expression.

### Evaluation of the prognostic model index

Based on the same formula, we calculated the risk score for each patient. Then, patients were divided into high-risk and low-risk groups according to the median risk score of MDS. After obtaining the prognostic model index for predicting the prognosis of UCEC patients, a series of measures were taken to evaluate the model.

**Fig. 4:**
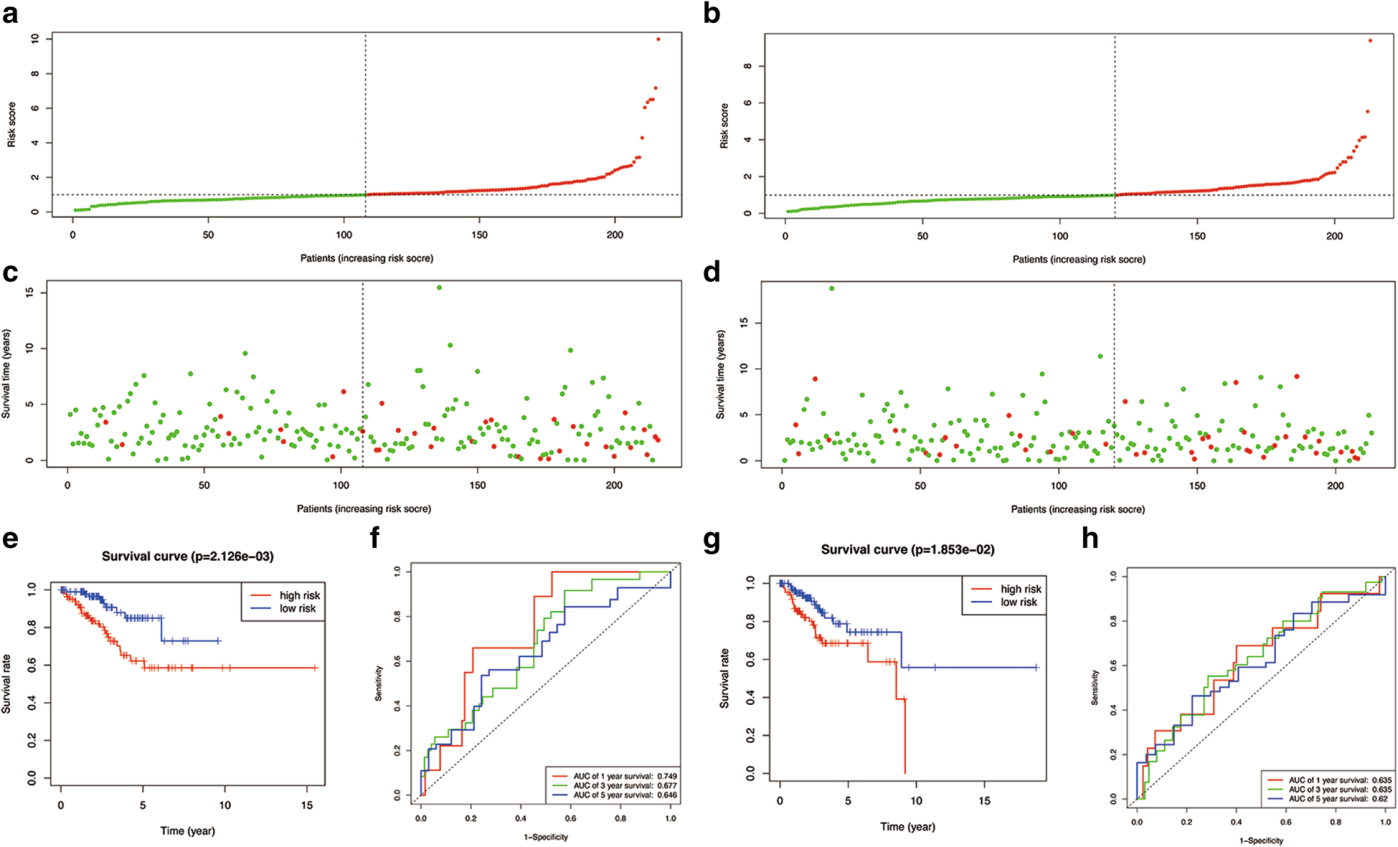
Prognostic analysis of the training cohort and the testing cohort. **A** Risk score distribution of patients in the prognostic model in training cohort. **B** Risk score distribution of patients in the prognostic model in testing cohort. **C** Survival status scatter plots for patients in the training cohort. **D** Survival status scatter plots for patients in the testing cohort. **E** Kaplan–Meier curve analysis in the training cohort. **F** Time-dependent ROC curve analysis in the training cohort. **G** Kaplan–Meier curve analysis in the testing cohort. **H** Time-dependent ROC curve analysis in the testing cohort

Firstly, the survival status of patients in the high-risk group and the low-risk group is shown in Fig. 4. Figure 4A, B show the result of risk classification of patients in training cohort and in testing cohort according to MDS respectively. From Fig. 4C, D we found that no matter in training cohort or in testing cohort, as the risk score increases, the number of dead patients increases. Kaplan–Meier curves based on the log-rank test were created to visualize the prognostic value of MDS in training cohort and in testing cohort. From Fig. 4E, G, we found that whether in training cohort or testing cohort, patients with high-risk score have a poor prognosis. Figure 4F is a time-dependent ROC curve in training cohort. The AUC values of the ROC curves in 1, 3, and 5 year were all > 0.60. Among them, MDS showed an excellent performance in predicting the 1-year survival rate of UCEC patients. Figure 4H is a time-dependent ROC curve in testing cohort. The AUC values in 1, 3, and 5 year were 0.635, 0.635 and 0.62 respectively.

Secondly, we further evaluated the prognostic value of MDS in patients with different clinical characteristics (Additional file 3: Figure S3). According to age, grade, pathological type and stage, we divided all patients into different groups. Kaplan–Meier curves were created in different groups. The prognostic model we constructed showed good prognostic ability in both patients ≤ 60 years and patients > 60 years (p = 0.002 and p = 0.022 respectively, Additional file 3: Figure S3A, B respectively). Additional file 3: Figure S3C, D show the survival curves of patients with grade 1–2 and patients with grade 3–4, respectively. Among them, MDS could better distinguish the survival in patients with grade 3 and grade 4 (p < 0.001). Additional file 3: Figure S3E reveals that MDS had a good prognostic ability in patients with endometrial cancer (p < 0.001), while seemed to be meaningless in other types of patients (p = 0.962, Additional file 3: Figure S3F). In patients with stage 1 to 2, MDS also showed good prognostic value (p = 0.020, Additional file 3: Figure S3G). However, in patients with stage 3 to 4, MDS was unable to distinguish patients with different survival outcomes (p = 0.090, Additional file 3: Figure S3H).

Thirdly, to further evaluate whether our model could be used as an independent prognostic factor, we included some key clinical characteristics containing age, stage, histological type, grade and MDS as independent variables. In training cohort, by means of univariate and multivariate Cox regression analysis, our established MDS remained significant (both *P* < 0.05, Table 2). While in entire cohort, stage, grade and MDS were regarded as independent prognostic factors.

**Table 2:** Cox regression analysis data

Variables	Training cohort				Entire cohort			
	Univariate Cox regression analysis		Multivariate Cox regression analysis		Univariate Cox regression analysis		Multivariate Cox regression analysis	
	p value	Hazard ratio	p value	Hazard ratio	p value	Hazard ratio	p value	Hazard ratio
Age	0.117	1.835 (0.859–3.919)			**0.023**	1.855 (1.088–3.162)	0.194	1.439 (0.830–2.494)
Stage	**0.003**	2.778 (1.424–5.418)	0.08	1.934 (0.923–4.050)	**< 0.001**	4.373 (2.733–6.996)	**< 0.001**	3.485 (2.148–5.653)
Histological type	**0.027**	2.156 (1.091–4.262)	0.828	0.912 (0.395–2.101)	**< 0.001**	2.955 (1.862–4.688)	0.133	1.500 (0.884–2.545)
Grade	**0.017**	2.766 (1.204–6.356)	0.1	2.153 (0.864–5.368)	**< 0.001**	3.715 (1.905–7.244)	**0.034**	2.202 (1.060–4.571)
MDS	**< 0.001**	1.391 (1.201–1.610)	**0.003**	1.289 (1.089–1.524)	**< 0.001**	1.284 (1.139–1.447)	**0.035**	1.154 (1.010–1.318)

Bold values indicate *p* < 0.05

What’s more, we once again verified the differences of key prognostic DMDGs between the high-risk group and the low-risk group from the gene expression levels and methylation levels in entire TCGA cohort (Additional file 4: Figure S4). Then, the relationship between the prognostic model we constructed (including MDS and 3 risk genes) and clinical characteristics was also revealed (Additional file 5: Figure S5).

**Fig. 5:**
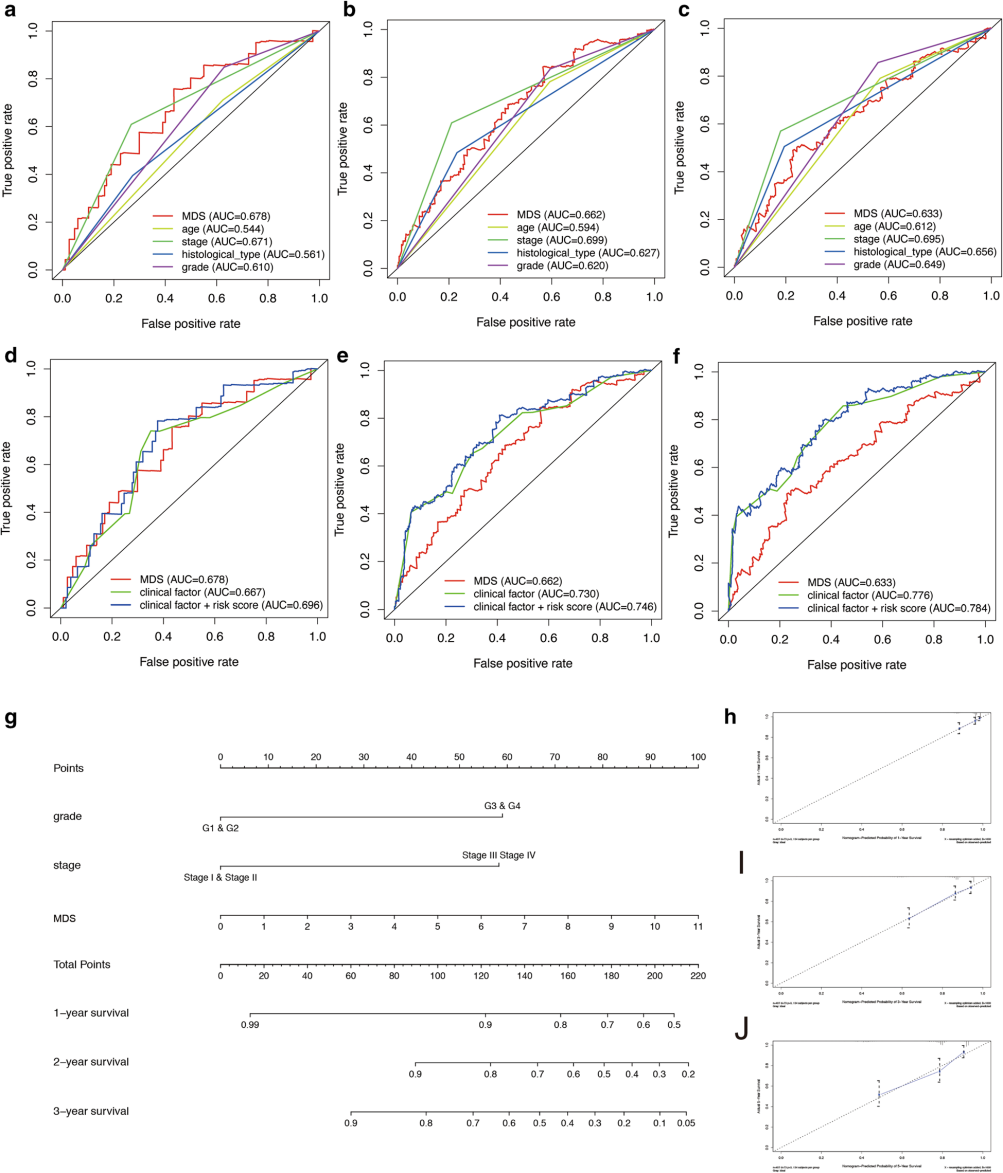
multivariate ROC curve analysis and nomogram with Calibration curves for the prediction of prognosis at 1, 3 and 5 years in the TCGA cohort. **A**, **B** ROC curve analysis with multiple variables for predicting 1-year OS. **C**, **D** ROC curve analysis with multiple variables for predicting 3-year OS. **E**, **F** ROC curve analysis with multiple variables for predicting 5-year OS. **G** Nomogram for OS. **H** Calibration curves at 1 year. **I** Calibration curves at 3 years. **J** Calibration curves at 5 years

Figure 5A–F show the time-dependent ROC curves of the multi-prognostic signatures. Figure 5A, B show the multivariate ROC curves in 1 year. Figure 5D shows the multivariate ROC curves in 3 years. Figure 5E, F show the multivariate ROC curves in 5 years. We found that MDS, stage and grade have similar accuracy in predicting the prognosis of UCEC patients. In addition, it was interesting that after combining MDS and clinical information into a mixed model, more accurate prediction capabilities could be obtained (Fig. 5B, D, F).

Text: “Figure 5D and Figure 5D” has been changed as “Figure 5D”. Please check and amend if any.we have checked it.Thanks.

### Construction of prognostic nomogram

Finally, to better predict the 1-year OS, 3-year OS and 5-year OS of UCEC patients, we constructed a new Nomogram based on the results of the multivariate Cox regression analysis of independent prognostic factors (Fig. 5G). Figure 5H–J show the Calibration curves of the nomogram for the probability of OS at 1, 3 and 5 year. The C-index of the nomogram for OS prediction was 0.764 (95% CI = 0.702–0.826). Additional file 6: Figure S6 shows the time-dependent C-index of MDS, stage, grade, and nomogram.

### Gene-set enrichment analysis

**Fig. 6:**
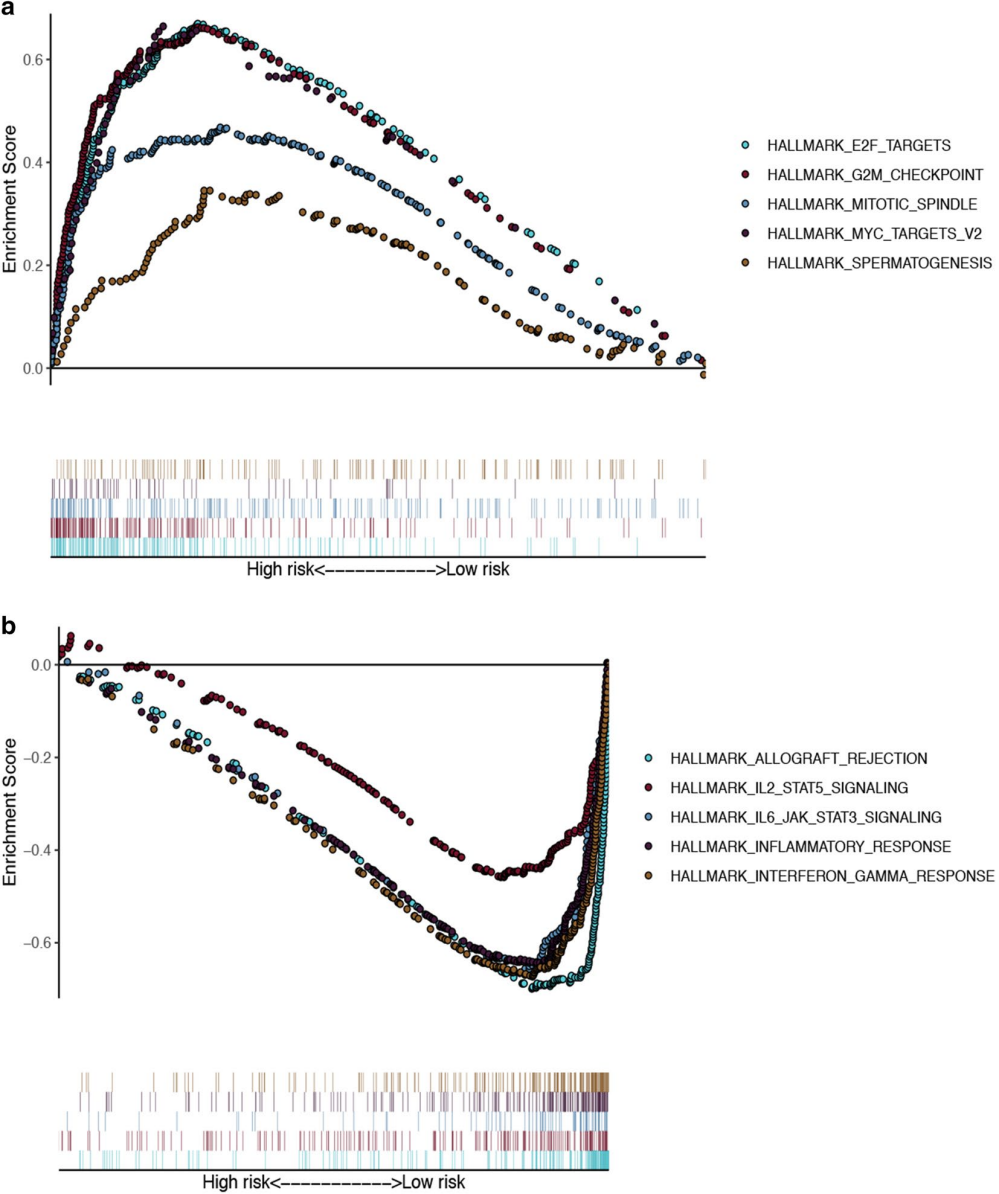
GSEA enrichment analysis. **A** Gene-set enrichment analysis of genes that are differentially expressed in high-risk group. **B** Gene-set enrichment analysis of genes that are differentially expressed in low-risk group

To uncover the biological pathways most likely to be related to MDS, we conducted a GSEA analysis (Fig. 6). Among them, E2F TARGETS, G2M CHECKPOINT, MITOTIC SPINDLE, MYC TARGETS V2 and SPERMATOGENESIS were found to be significantly enriched in high-risk group. While in low-risk group, ALLOGRAFT REJECTION, IL2 STAT5 SIGNALING, IL6 JAK STAT3 SIGNALING, INFLAMMATORY RESPONSE and INTERFERON GAMMA RESPONSE were significantly enriched.

### TMB, mRNAsi and m6A regulators in two groups

Through GSEA analysis, we found an interesting phenomenon that some immune-related pathways may affect the prognosis of UCEC. Which caused us to think, was the prognosis of UCEC patients related to the difference in the immune microenvironment within the tumor? It has been reported that the stemness of tumors, the methylation modification mediated by m6A regulatory factors, and TMB are related to the immune microenvironment of tumors [29, 30]. Therefore, we decided to further explore whether there were differences between the two groups of patients in these aspects.

**Fig. 7:**
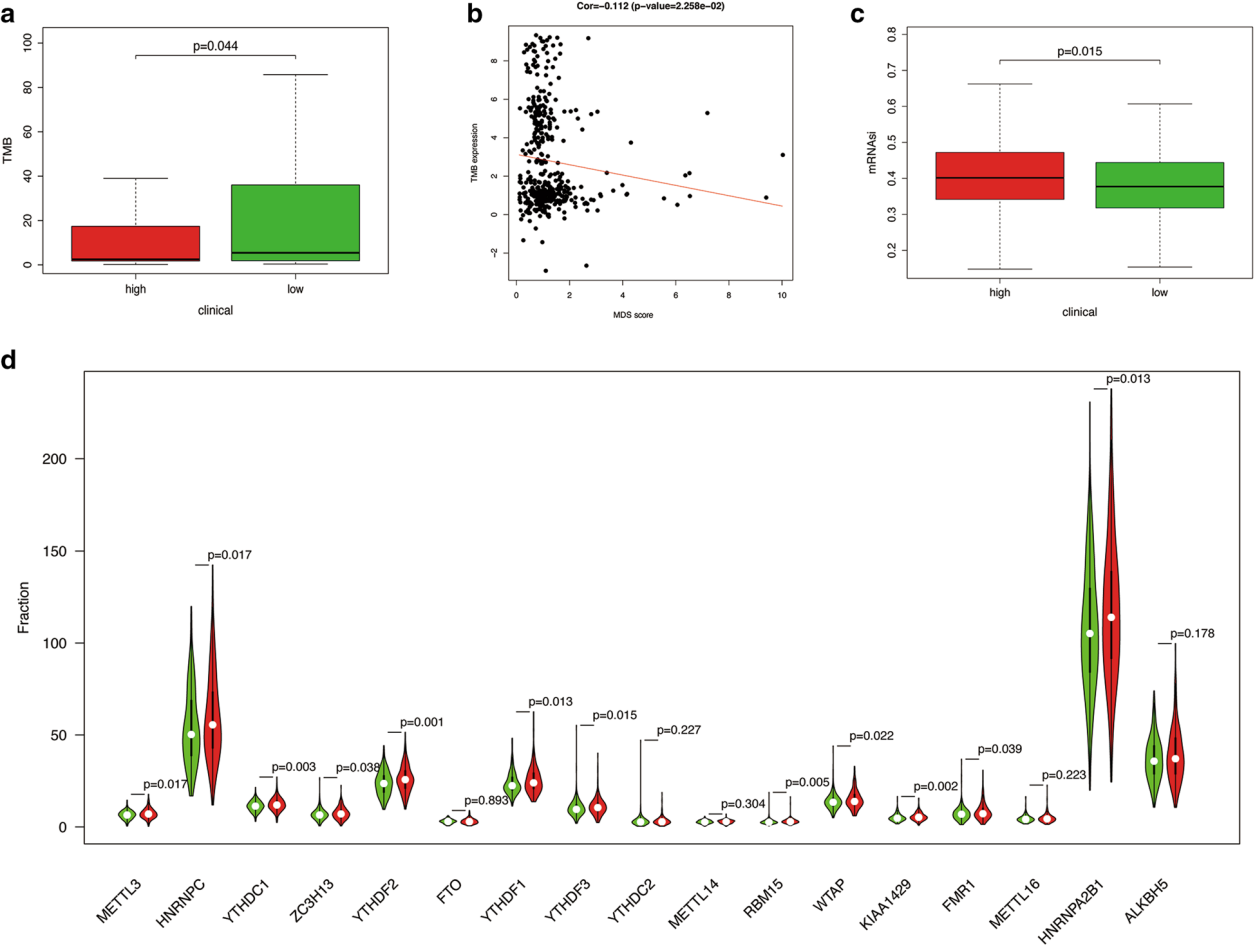
The correlation between MDS and other phenotypic studies. **A**, **B** The correlation between MDS and tumor mutation burden (TMB). **C** The correlation between MDS and stem cell index (mRNAsi). **D** The correlation between MDS and gene expression of m6A regulators. Green represents the low-risk group, and red represents the high-risk group

From Fig. 7A, B, we could find that patients in the low-risk group have higher TMB level than patients in the high-risk group. There was a significant negative correlation between TMB and MDS. The somatic mutations seen in the two groups are visually displayed in Additional file 7: Figure S7A, B. Additional file 7: Figure S7C suggests that TP53 has a higher mutation frequency in the high-risk group, while ARID1A, CTCF, KMT2B, MUC16 and PTEN have a higher mutation frequency in the low-risk group. In addition, the mRNAsi level of the high-risk group was significantly higher than that of the low-risk group (Fig. 7C). What’s more, there were significant differences in the expression of some m6A regulatory factors between patients in high-risk group and patients in low-risk group (Fig. 7D).

### IPS and the expression of immune check point in two groups

**Fig. 8:**
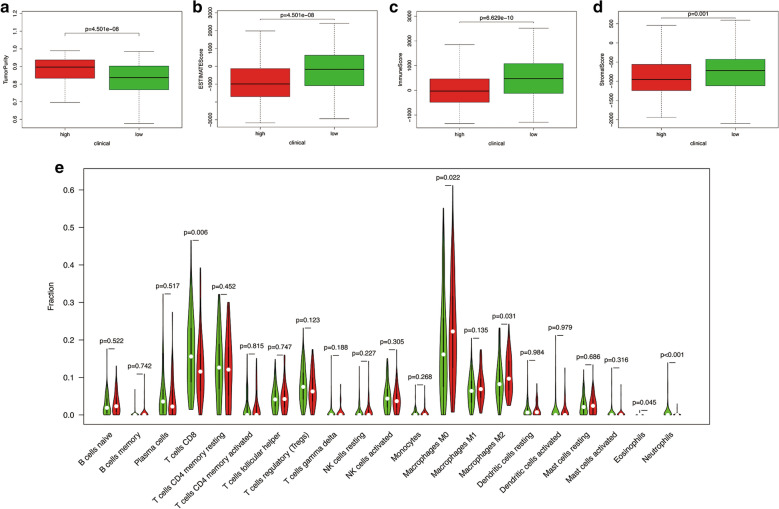
The correlation between MDS and immune related indicators. **A** The correlation between MDS and tumor purity. **B** The correlation between MDS and ESTIMATEScore. **C** The correlation between MDS and ImmuneScore. **D** The correlation between MDS and StromalScore. **E** The correlation between MDS and immune cell infiltration

Through the ESTIMATE evaluation method, TumorPurity, ImmuneScore and StromalScore were calculated. Figure 8 indicates that patients in the low-risk group have lower TumorPurity and higher ImmuneScore and StromalScore. Figure 8E shows the correlation between MDS and immune cell infiltration. From which, we could find that compared with patients in high-risk group, patients in low-risk group had more T cells CD8, Eosinophils and Neutrophils, while the Macrophages M0 and Macrophages M2 was at low level. Furthermore, the score of macrophage M2 was found to be significantly positively correlated with MDS. While the score of T cells CD8 was found to be significantly negatively correlated with MDS (Additional file 8: Figure S8).

Then, the ssGSEA algorithm was used to quantify the level of immune infiltration. Comparison of the ssGSEA scores between different risk groups is shown in Additional file 9: Figure S9. From which we found that in addition to score of NK cells and score of type II IFN response, other parameters of immune cell infiltration indicate that patients in the low-risk group have higher immune activity.

**Fig. 9:**
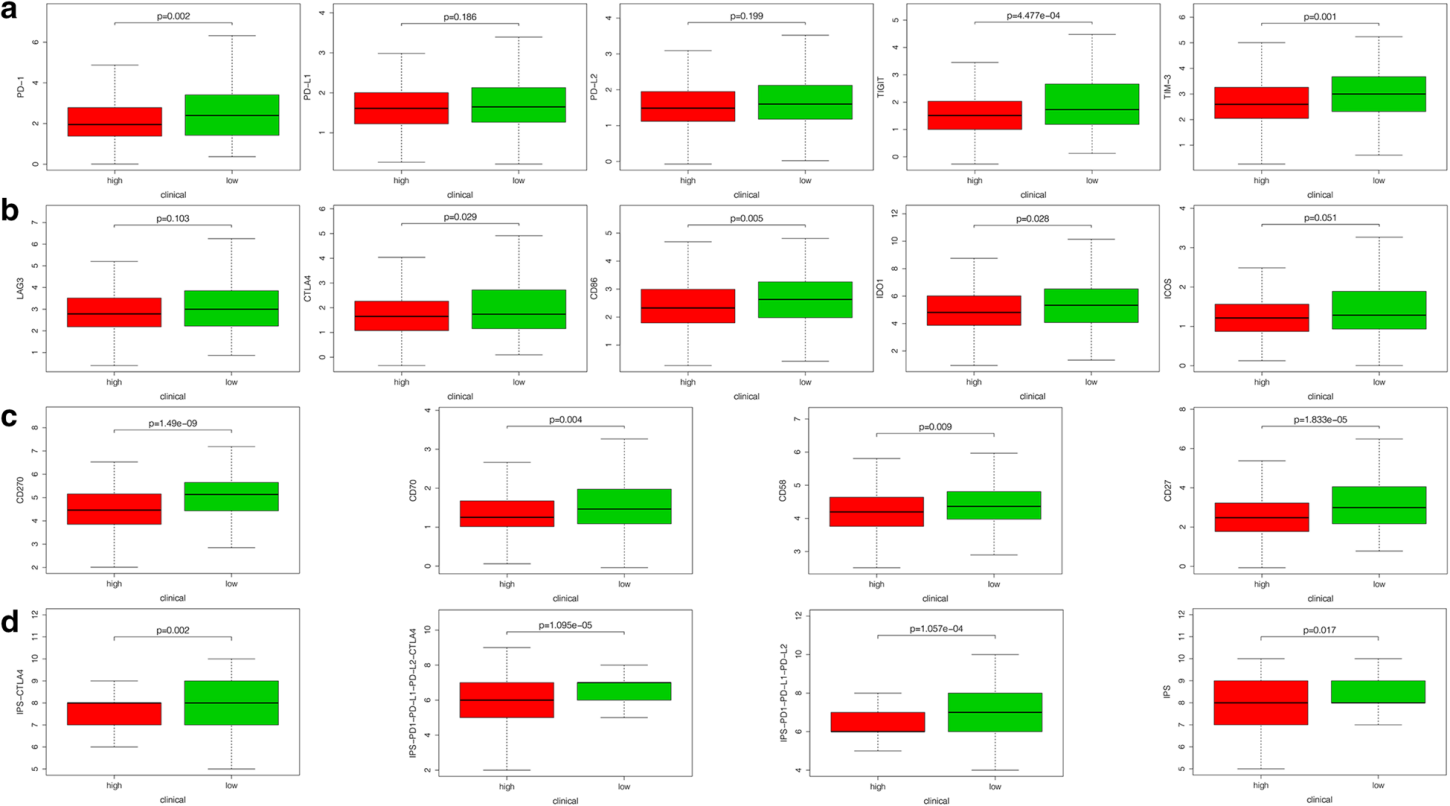
The correlation between MDS and gene expression of immune checkpoints. **A**–**C** The relationship between MDS and 12 immune checkpoint molecules. **D** The correlation between MDS and immunophenoscore (IPS)

To further explore whether MDS can be instructive in guiding immunotherapy, we compared the expression of immune checkpoints and immunophenoscore between two groups (Fig. 9). The results showed that 12 immune checkpoint molecules were highly expressed in patients in the low-risk group (Fig. 9A–C). Furthermore, the gene expression of these immune checkpoints was found to be significantly negatively correlated ted with MDS (Additional file 10: Figure S10). In addition, similar results were obtained in terms of immunogenicity. The IPS, IPS-CTLA4, IPS-PD1-PD-L1-PD-L2 and IPS-PD1-PD-L1-PD-L2-CTLA4 scores were higher in patients in the low-risk group (Fig. 9D).

### Joint survival analysis

**Fig. 10:**
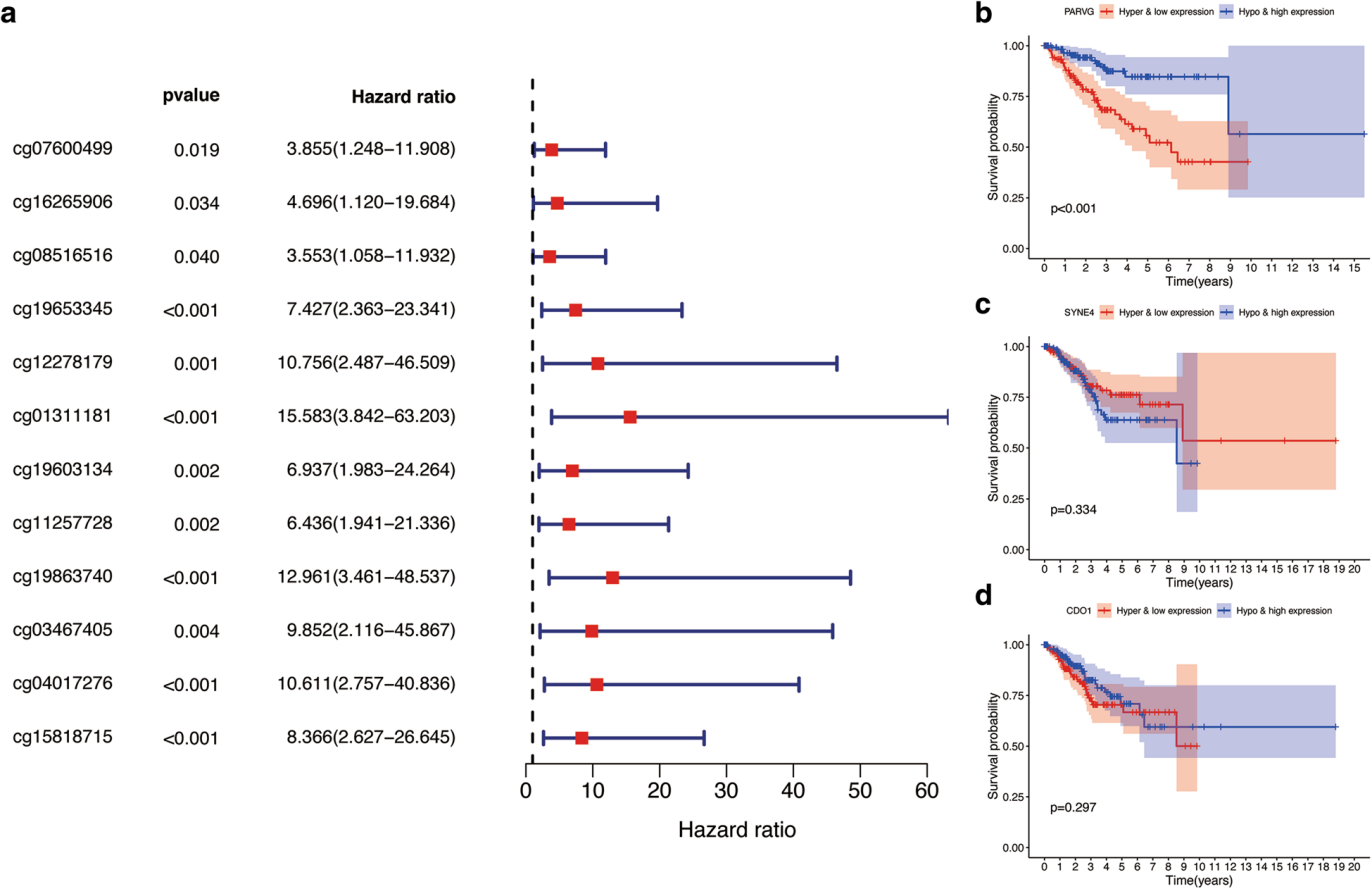
Joint survival analysis. **A** Univariate Cox regression analysis assessing the relationship between methylation sites of 3 key MDMGs and overall survival in TCGA-UCEC. **B**–**D** Meaningful Kaplan–Meier survival curves for the methylation-driven genes and methylation joint survival analysis

According to the results of correlation analysis, we finally determined 21 methylation sites (the methylation level of 11 methylation sites is negatively correlated with the expression level of CDO1, and the methylation level of 9 methylation sites is negatively correlated with the expression level of PARVG. and the level of methylation at a methylation site is negatively correlated with the expression level of SYNE4) (Additional file 11: Figure S11). The results of the univariate Cox regression analysis showed that 12 of methylation sites significantly related to CDO1, PARVG and SYNE4 were associated with positive survival outcomes (with all HR > 1) (Fig. 10A). After obtaining mixed models that integrating methylation level and gene expression, we conducted survival analysis on them. The results can be found in Fig. 10B–D. Which shows that hypermethylation and low gene expression PARVG was significantly related to the negative prognosis of UCEC patients. However, the expression of SYNE4 and CDO1 were not related to the prognosis of UCEC patients.

## Discussion

Uterus corpus endometrial cancer is one of the main gynecological cancers, its biological, clinical, histological and genetic characteristics are significantly heterogeneous. The traditional classification of endometrial cancer cannot fully reflect the heterogeneity of tumors. Therefore, there are certain limitations in predicting the prognosis of patients and the effect of treatment response. In recent years, more and more studies have provided the basis for prognostic judgment and clinical treatment of UCEC. In 2013, the American Cancer Genome Map Research Center for Endometrial Cancer provided revolutionary insights into the classification of endometrial cancer. According to the integrated genome characteristics, they reclassified endometrial cancer into 4 different types, each with different clinical outcomes. This classification provides more accurate information for clinical treatment and prognosis judgment. What’s more, it is recommended by NCCN guidelines to effectively predict the prognosis of UCEC [31]. Using the miRNA data of endometrial cancer in TCGA, Luo et al. created a prediction model for overall survival and recurrence-free survival, which can effectively predict the prognosis of endometrial cancer patients [32]. However, few evidence has suggested that alterations in DNA methylation could lead to tumor progression and local immunosuppression in the TME.

As we all know, abnormal DNA methylation is a major epigenetic event that promotes the development and progression of cancer. Abnormal DNA methylation is not only related to the shutdown of tumor suppressor genes, but also closely related to the tumor’s malignancy and prognosis [33]. Therefore, our article aims to discover new DNA methylation-driven biomarkers to more accurately predict the prognosis of UCEC, and to explore the possible mechanisms of DNA methylation-driven biomarkers differences between patients with different prognosis. In terms of methods, we used MethylMix to combine the methylation data with the mRNA data from TCGA-UCEC and perform correlation analysis [34]. After that 48 methylation-driven genes were found to be significantly differentially expressed. Then, TCGA-UCEC was randomly divided into training cohort and testing cohort according to the distribution of clinical characteristics, which were used to train and verify the prediction model. We further used univariate Cox regression analysis, multivariate Cox regression analysis and Lasso Cox regression analysis to filer prognosis related genes. 3 DMDGs were finally identified to construct an independent prognostic index. After that, we verified this model in the testing cohort, the results showed that this model could be used as an independent prognostic factor and this model had a good ability to predict the prognosis of UCEC patients. Furthermore, we combined MDS with prognostic clinical indicators to construct a new prognostic nomogram. On the one hand, the results of ROC curve analysis, survival curve analysis and Cox regression analysis all show that MDS can accurately distinguish UCEC patients with different survival outcomes. On the other hand, it is interesting that MDS is positively correlated with some known poor prognostic clinical features (old age, high grade, pathological type with high malignancy and high stage). Therefore, the prognostic model we constructed has a high degree of credibility to a certain extent.

PARVG, SYNE4 and CDO1 are the three risk DNA methylation-driven differential genes we identified. Among them, we verified the difference in expression of PARVG and SYNE4 in 13 pairs of normal tissues and cancer tissues. However, CDO1 was not differentially expressed between 13 pairs of normal tissues and cancer tissues. According to the large sample data showed in Additional file 4: Figure S4, the methylation level of CDO1 is lower mRNA expression of CDO1 is higher in the normal tissue than that in the tumor tissue. However, the range of the mRNA expression is much wide. When the number of the samples was limited like 13 pairs sample in our manuscript. The difference between groups will not be so significant. This might be the reason for the inconsistency between the qRT-PCR results.

PARVG, located in the q13.31 region of chromosome 22, has been proven not to be a tumor suppressor gene related to the occurrence and development of CRC and breast cancer [35]. The results of a methylation sequencing study on adenoid cystic carcinoma found that PARVG in tumor tissues showed lower methylation levels [36]. In addition, a report showed that the high expression of the PARVG gene caused by low-level DNA methylation is significantly related to the poor prognosis of renal cancer patients [37]. In our study, PARVG also showed consistent prognostic performance in UCEC (HR = 42.85, p < 0.05). SYNE4 is a member of the nesprin family of genes, that encode KASH (Klarsicht, Anc-1, Syne Homology) domain-containing proteins. SYNE4 is believed to be involved in the formation of the nucleus skeleton, but its role in tumors is still unknown. As for CDO1, it is the abbreviation of Cysteine Dioxygenase Type 1. CDO1 is a putative tumor suppressor gene in human cancers, and its abnormal DNA methylation level has been studied in a variety of tumors. In gastric cancer, researchers found that CDO1 has significantly DNA hypermethylation than normal tissues. More interestingly, the higher the DNA methylation level of CDO1 in gastric cancer patients, the better the survival prognosis of patients receiving chemotherapy [38]. In lung cancer, researchers also found a similar pattern that the promoter of CDO1 in tumor tissues is hypermethylated. Also, the higher the hypermethylation frequency of CDO1, the lower the degree of tumor differentiation [39]. Which may be related to the limit of the futile metabolism of cysteine and the consumption of cell NADPH caused by CDO1 being silenced, which promotes the proliferation of NSCLC [40]. In pancreatic ductal adenocarcinoma, the hypermethylation of the CDO1 promoter is highly specific in tumor tissues. This feature has been successfully used by researchers as a potential diagnostic biomarker [41]. In our study, the low expression of CDO1 driven by DNA hypermethylation is also significantly related to the poor prognosis of UCEC. Therefore, judging from the genetic makeup of MDS, the predictive prognosis model we constructed is highly reliable.

In addition to constructing a nomogram with high application prospects to accurately predict the prognosis of UCEC, we also explored the possible mechanisms that cause different survival outcomes among UCEC patients through GSEA. Which suggested that there were some changes in immune-related pathways between the high-risk group and the low-risk group. we could find that compared with patients in high-risk group, patients in low-risk group had more T cells CD8, Eosinophils and Neutrophils, while the Macrophages M0 and Macrophages M2 were at low level. Furthermore, the score of macrophages M2 was found to be significantly positively correlated with MDS. While the score of T cells CD8 was found to be significantly negatively correlated with MDS*.* As early as 2018, Zhang et al. proposed that macrophages induce PDPK1-mediated PGK1 phosphorylation in tumor cells by secreting IL6 to regulate the direction of PGK1 catalytic reaction, thereby enhancing the glycolysis of tumor cells and ultimately promoting the malignant progression of the tumor [42]. T cells CD8+ are the main effector cells that perform antigen-specific killing of tumor cells. Many reports have pointed out that targeted intervention in the glucose metabolism pathway of T cells CD8+ can effectively enhance their anti-tumor function. At the same time, we also explored the differences between the two groups in terms of immunity, RNA methylation modification (m6A), tumor stemness (mRNAsi), and tumor mutation burden (TMB). We found an interesting phenomenon that MDS has a clear relationship with immune-related events. Which was manifested in the significant negative correlation between MDS and TMB (cor = − 0.112, p < 0.05). The high tumor mutation burden (TMB) (TMB ≥ 10 Muts/Mb) has been approved by the US Food and Drug Administration (FDA) to guide biomarkers for immunotherapy. TMB was defined as the number of somatic variants per megabase of genome. These somatic mutations potentially result in new or fragmented proteins/peptides, which can be recognized as exogenous, and elicit an antitumor immune response. TMB can predict the efficacy of immunotherapy outcomes, and filtered candidate patients for immunotherapy treatment. However, in clinical practice, the TMB was assessed by whole-genome sequencing (WGS), whole-exome sequencing (WES) and targeted panel sequencing. Due to the high cost, relatively long turnaround time and the need for sufficient tissue samples. Evaluation of TME in clinical practice is not feasible. In this research, we found the closely relationship between MDS and TMB. With the help of this MDS, we can indirectly reflect the TMB status and provide valuable information on the results of immunotherapy response. The ssGSEA algorithm was also used by us to quantify the condition of immune infiltration. Interestingly, in addition to score of NK cells and score of type II IFN response, other parameters of immune cell infiltration indicate that patients in the low-risk group have higher immune activity. In addition, the expression of some immune checkpoints in patients with high MDS was significantly lower than that of patients with low MDS. The low expression of some immune checkpoints in high-risk group may cause by the low fraction of CD8+ T cells. Which was consist with the TMB results. Therefore, the MDS we constructed can not only accurately predict the prognosis of UCEC patients, but also accurately reflect their tumor immune microenvironment. What’s more, patients in the high-risk group had lower IPS. Which means that the results of our research also provide a reference method for guiding UCEC’s immunotherapy.

However, our study still has some limitations: the results of our study were only validated in the TCGA database. The prognostic model requires more data support from clinical patients. In addition, the mechanism by which DNA methylation-driven genes affect the prognosis of patients with UCEC needs to be further explored through in vivo and in vitro experiments.

## Conclusion

All in all, we have successfully established a risk model (MDS) based on 3 DMDGs, which could accurately predict the prognosis of patients with UCEC. A nomogram combining clinical variables and MDS was also drawn to improve the accuracy of the prediction. In addition, our risk score model could connect with Methylation-Driven genes and tumor immune microenvironment, which provides a comprehensive perspective for clarifying the underlying mechanisms that determine the prognosis for UCEC. Our research may shed new light on UCEC patient’ prognosis and treatment management. However, further experiments are also required to validate our findings.

## Supplementary Information

**Additional file 1: Figure S1.** Flow diagram of the study.

**Additional file 2: Figure S2.** Construction of MDS in patients with UCEC. (A) Elucidation for LASSO coefficient profiles of prognostic DMDGs. (B) Validation was performed for tuning parameter selection through the LASSO regression model. (C) qRT-PCR results of CDO1 in tissue samples. (D) qRT-PCR results of SYNE4 in tissue samples. (E) qRT-PCR results of PARVG in tissue samples.

**Additional file 3: Figure S3.** Prognostic analysis of the entire cohort. (A) Kaplan–Meier curve analysis in patients ≤ 60 years old. (B) Kaplan–Meier curve analysis in patients > 60 years old. (C) Kaplan–Meier curve analysis in patients with grade 1 and grade 2. (D) Kaplan–Meier curve analysis in patients with grade 3 and grade 4. (E) Kaplan–Meier curve analysis in patients with endometrial cancer. (F) Kaplan–Meier curve analysis in patients with mixed and serious Pathological type. (G) Kaplan–Meier curve analysis in patients with stage I and stage II. (H) Kaplan–Meier curve analysis in patients with stage III and stage IV.

**Additional file 4: Figure S4.** Differences in expression and methylation of key genes between high-risk and low-risk groups. (A) The difference of CDO1 gene expression between the two groups. (B) The difference of CDO1 DNA methylation level between the two groups. (C) The difference of SYNE4 gene expression between the two groups. (D) The difference of SYNE4 DNA methylation level between the two groups. (E) The difference of PARVG gene expression between the two groups. (F) The difference of PARVG DNA methylation level between the two groups. * means p < 0.05. ** means p < 0.01. *** means p < 0.001.

**Additional file 5: Figure S5.** The relationship between the prognostic model we constructed (including 3 risk genes and MDS) and clinical characteristics. (A) The relationship between MDS and age. (B) The relationship between MDS and Grade. (C) The relationship between MDS and histological type. (D) The relationship between MDS and Stage. (E) The relationship between PARVG gene expression and age. (F) The relationship between PARVG gene expression and histological type. (G) The relationship between PARVG gene expression and stage. (H) The relationship between SYNE4 gene expression and age. (I) The relationship between SYNE4 gene expression and histological type. (J) The relationship between SYNE4 gene expression and stage.

**Additional file 6: Figure S6.** The time-dependent C-index of MDS, stage, grade, and nomogram.

**Additional file 7: Figure S7.** Mutational landscape of UCEC tumor samples in high- and low-risk groups. (A) Waterfall plot representing the mutational landscape of the top 10 most frequently mutated genes in high-risk. (B) Landscape of the top 10 most frequently mutated genes in high-risk and low-risk group. (C) Forest plot illustrating the genes that exhibit significant differences in mutational rate between high- and low-risk UCEC samples groups with the threshold of p < 0.05.

**Additional file 8: Figure S8.** Correlation between MDS and immune cell infiltration. (A) The association between MDS and macrophage M2. (B) The association between MDS and T cells CD8. (C) The association between MDS and each type of immune cell.

**Additional file 9: Figure S9.** Comparison of the ssGSEA scores between different risk groups in the TCGA cohort. (A) The scores of 16 immune cells. (B) The scores of 13 immune-related functions. Red means high-risk group. Blue means low-risk group.

**Additional file 10: Figure S10.** Correlation between MDS and the expression of checkpoints. (A–L) The association between MDS and each checkpoint. (M) The landscape of the association between MDS and some immune checkpoint regulators.

**Additional file 11: Figure S11.** Correlation between methylation sites and the expression of 3 DMDGs. (A, B) The methylation level of 11 methylation sites is negatively correlated with the expression level of CDO1. (C, D) methylation level of 9 methylation sites is negatively correlated with the expression level of PARVG. (E) The level of methylation at a methylation site is negatively correlated with the expression level of SYNE4.

**Additional file 12: Table S1.** Clinicopathologic characteristics of patients with TCGA-UCEC.

**Additional file 13: Table S2.** Primers’ information.

## Data Availability

All the data used to support the findings of this study are included within the article. Please contact author for data requests.

## Abbreviations

UCEC: Uterus corpus endometrial cancer
MDGs: Differential methylation-driven genes
IPS: Immunophenoscore
GO: Gene ontology
KEGG: Kyoto Encyclopedia of Genes and Genomes
PI: Prognostic index
TMB: Tumor mutational burden
GSEA: Gene-set enrichment analysis
OS: Overall survival
ROC: Receiver operating characteristic
