# Supplementary figures and images for: Identification of methylation-driven genes prognosis signature and immune microenvironment in uterus corpus endometrial cancer
Source: Cancer Cell Int. 2021 Jul 10;21:365. doi: 10.1186/s12935-021-02038-z (PMC8272318; doi:10.1186/s12935-021-02038-z)

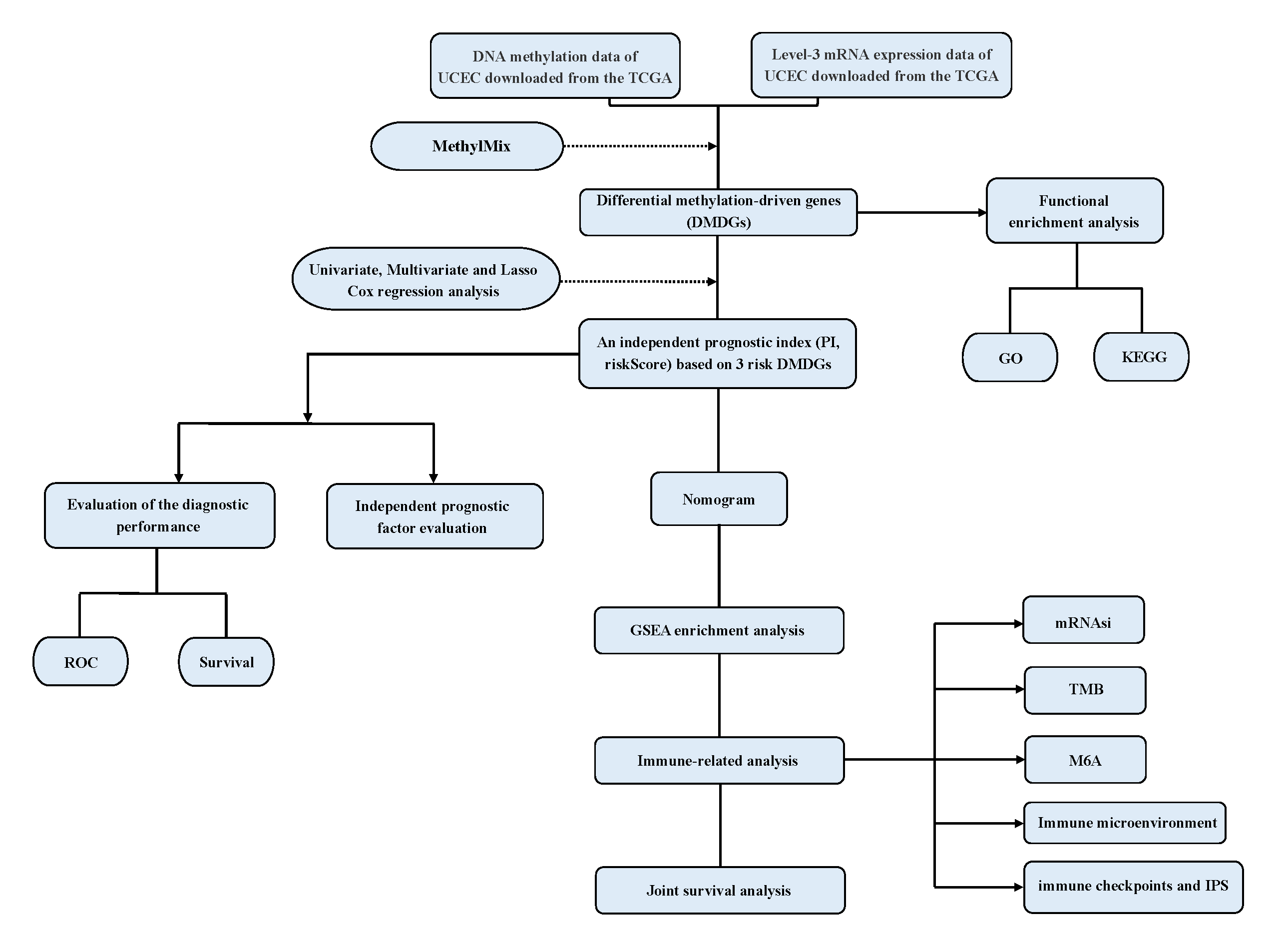

Supplement: Supplementary file 1 — Additional file 1: Figure S1. Flow diagram of the study. [file 12935_2021_2038_MOESM1_ESM.tiff]

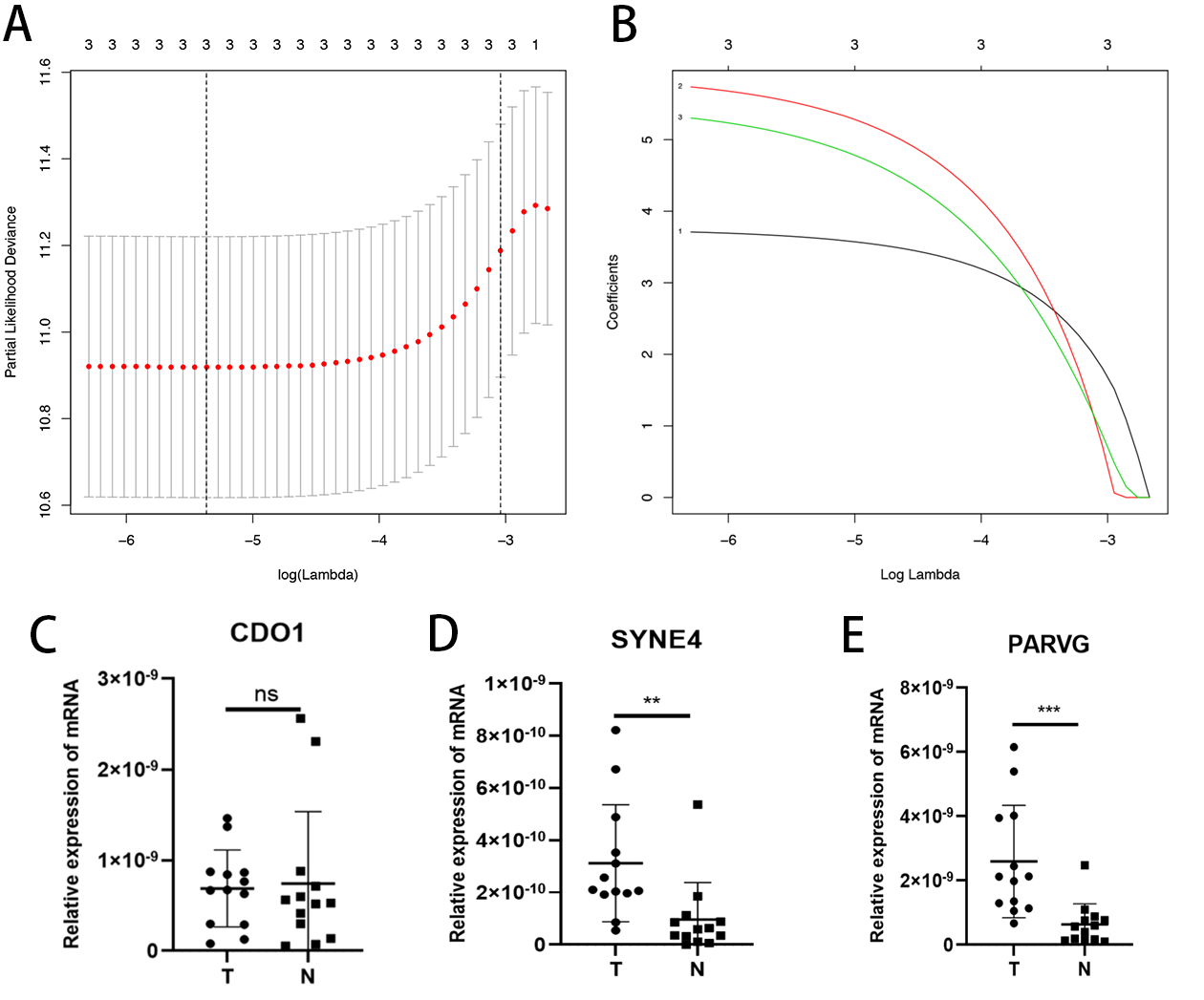

Supplement: Supplementary file 2 — Additional file 2: Figure S2. Construction of MDS in patients with UCEC. (A) Elucidation for LASSO coefficient profiles of prognostic DMDGs. (B) Validation was performed for tuning parameter selection through the LASSO regression model. (C) qRT-PCR results of CDO1 in tissue samples. (D) qRT-PCR results of SYNE4 in tissue samples. (E) qRT-PCR results of PARVG in tissue samples. [file 12935_2021_2038_MOESM2_ESM.tif]

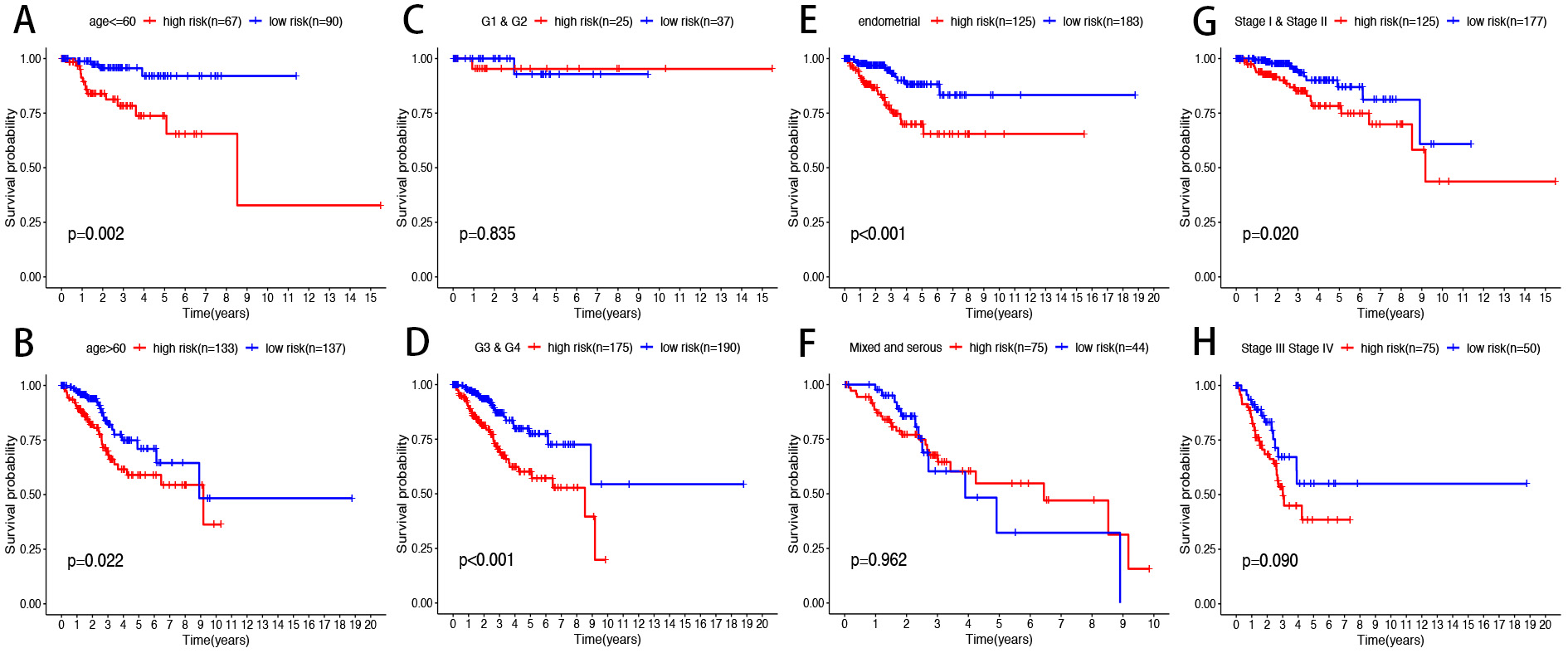

Supplement: Supplementary file 3 — Additional file 3: Figure S3. Prognostic analysis of the entire cohort. (A) Kaplan–Meier curve analysis in patients ≤ 60 years old. (B) Kaplan–Meier curve analysis in patients > 60 years old. (C) Kaplan–Meier curve analysis in patients with grade 1 and grade 2. (D) Kaplan–Meier curve analysis in patients with grade 3 and grade 4. (E) Kaplan–Meier curve analysis in patients with endometrial cancer. (F) Kaplan–Meier curve analysis in patients with mixed and serious Pathological type. (G) Kaplan–Meier curve analysis in patients with stage I and stage II. (H) Kaplan–Meier curve analysis in patients with stage III and stage IV. [file 12935_2021_2038_MOESM3_ESM.jpg]

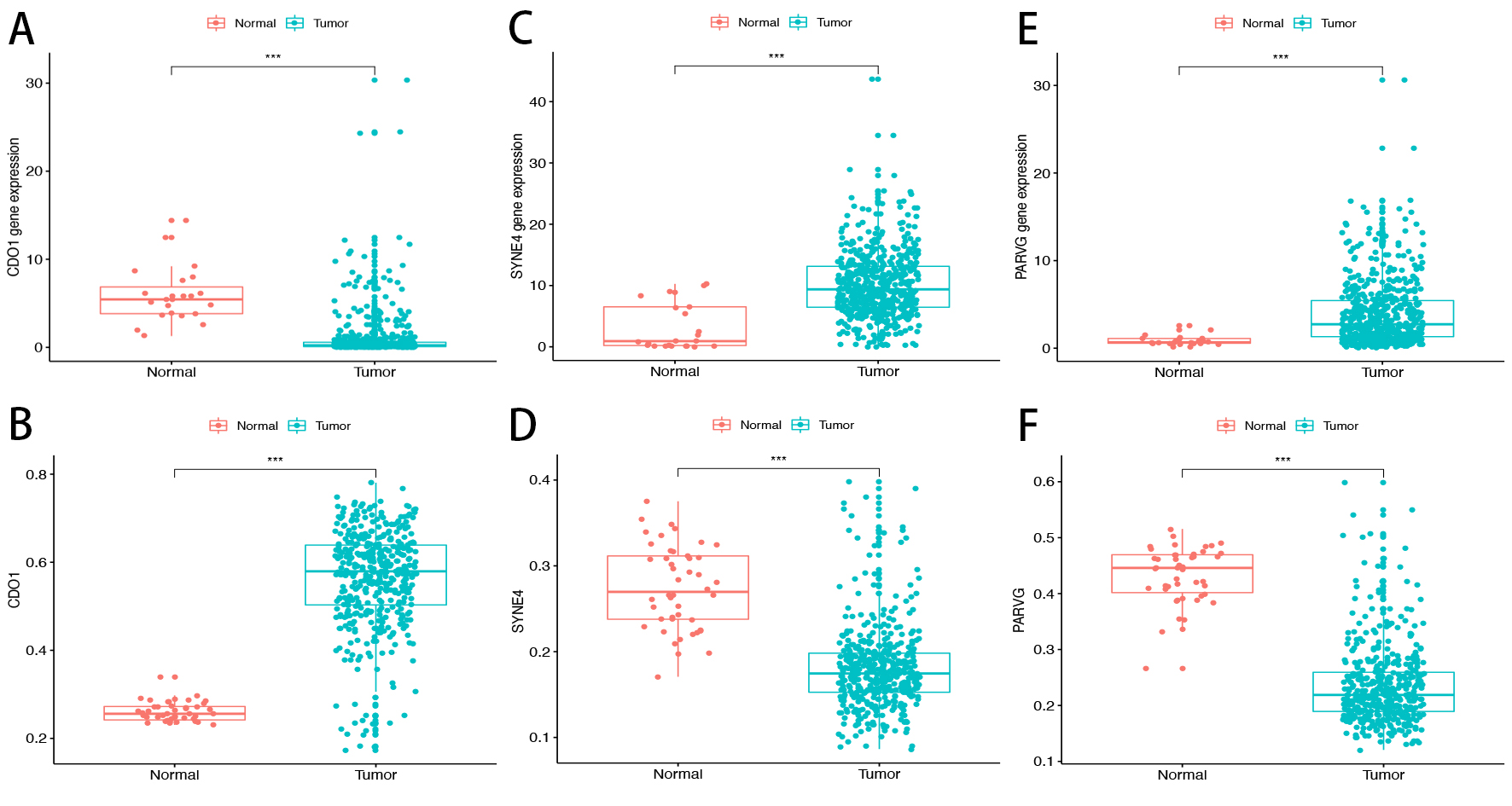

Supplement: Supplementary file 4 — Additional file 4: Figure S4. Differences in expression and methylation of key genes between high-risk and low-risk groups. (A) The difference of CDO1 gene expression between the two groups. (B) The difference of CDO1 DNA methylation level between the two groups. (C) The difference of SYNE4 gene expression between the two groups. (D) The difference of SYNE4 DNA methylation level between the two groups. (E) The difference of PARVG gene expression between the two groups. (F) The difference of PARVG DNA methylation level between the two groups. * means p < 0.05. ** means p < 0.01. *** means p < 0.001. [file 12935_2021_2038_MOESM4_ESM.jpg]

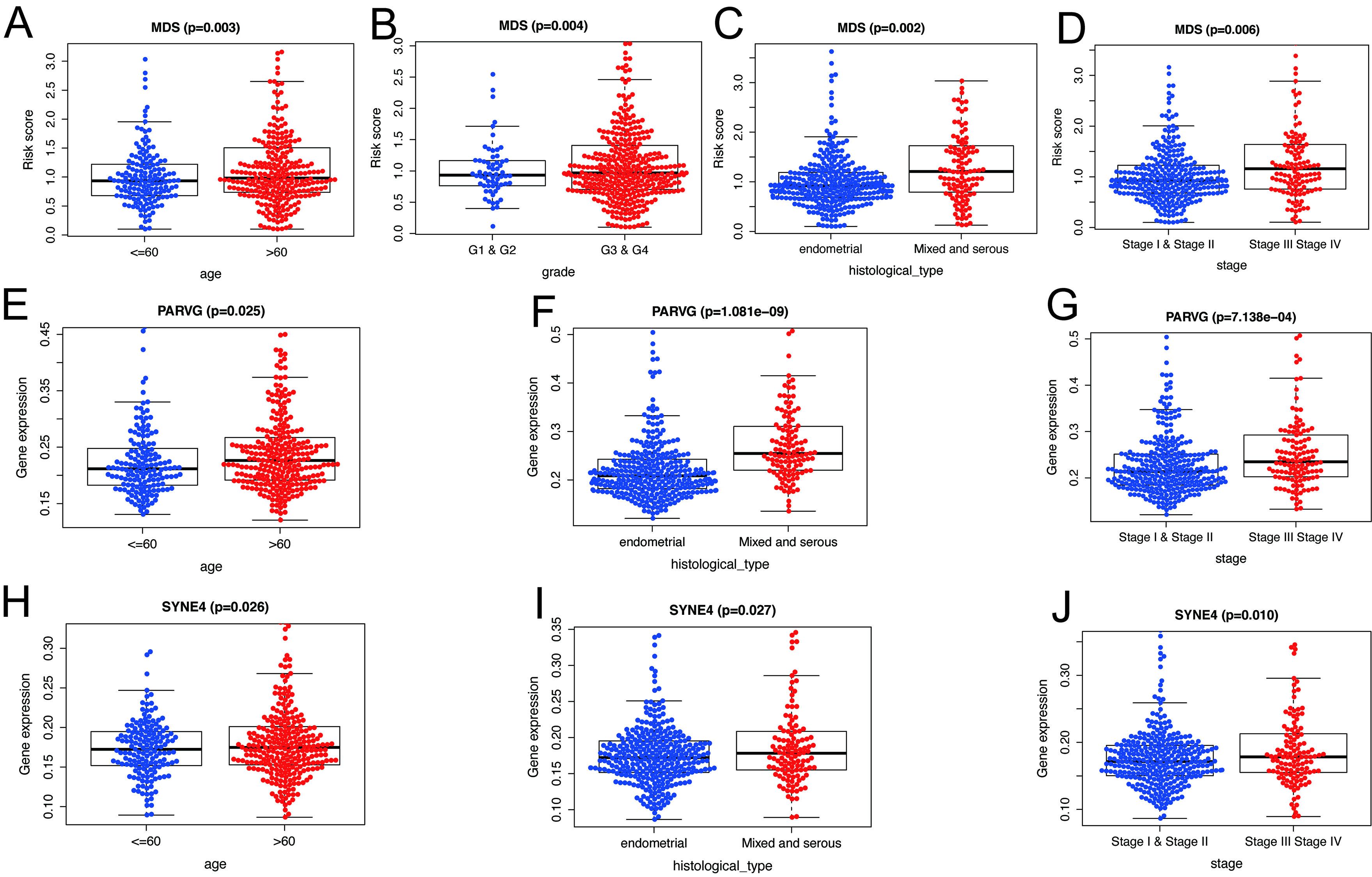

Supplement: Supplementary file 5 — Additional file 5: Figure S5. The relationship between the prognostic model we constructed (including 3 risk genes and MDS) and clinical characteristics. (A) The relationship between MDS and age. (B) The relationship between MDS and Grade. (C) The relationship between MDS and histological type. (D) The relationship between MDS and Stage. (E) The relationship between PARVG gene expression and age. (F) The relationship between PARVG gene expression and histological type. (G) The relationship between PARVG gene expression and stage. (H) The relationship between SYNE4 gene expression and age. (I) The relationship between SYNE4 gene expression and histological type. (J) The relationship between SYNE4 gene expression and stage. [file 12935_2021_2038_MOESM5_ESM.tif]

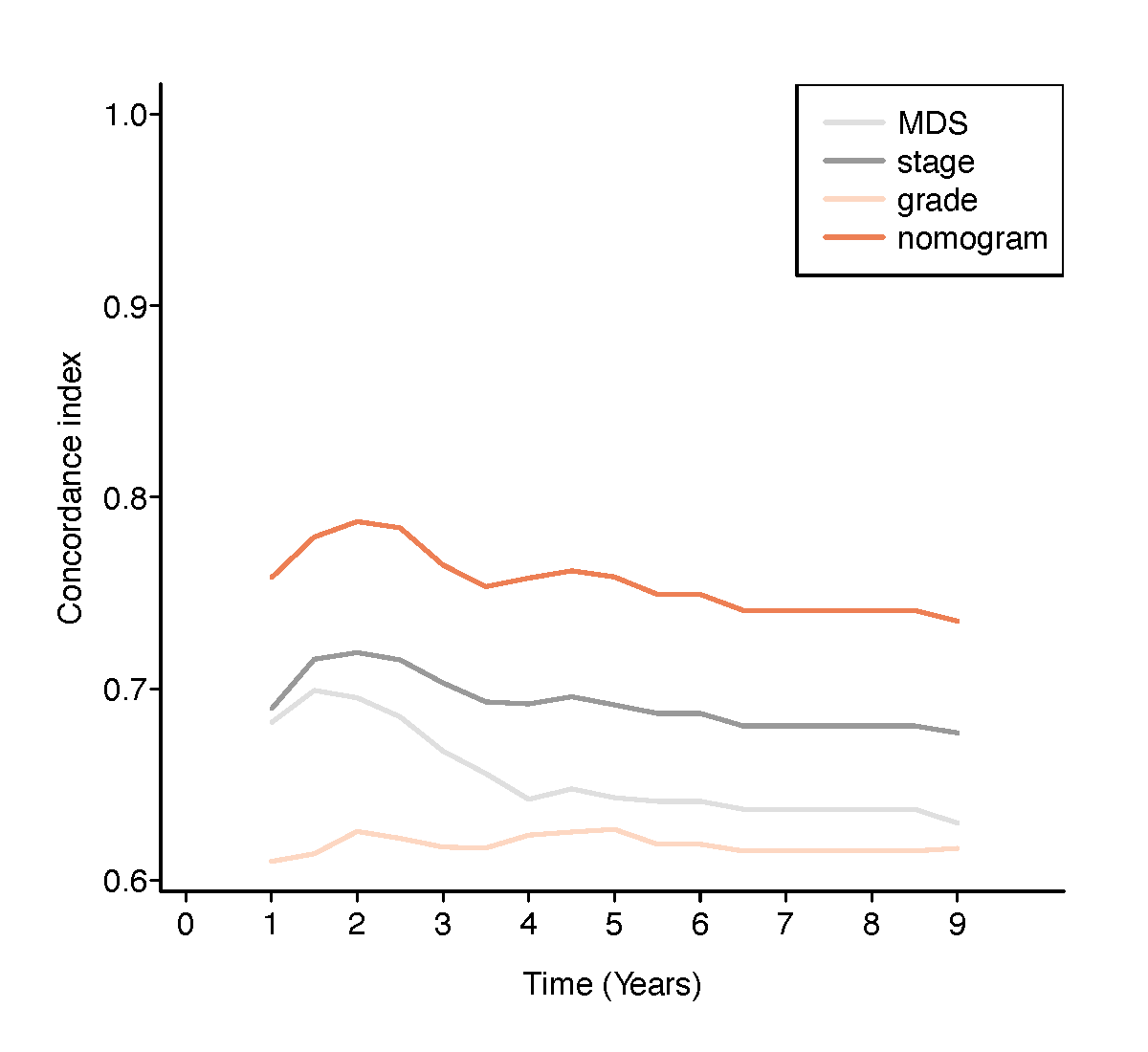

Supplement: Supplementary file 6 — Additional file 6: Figure S6. The time-dependent C-index of MDS, stage, grade, and nomogram. [file 12935_2021_2038_MOESM6_ESM.tiff]

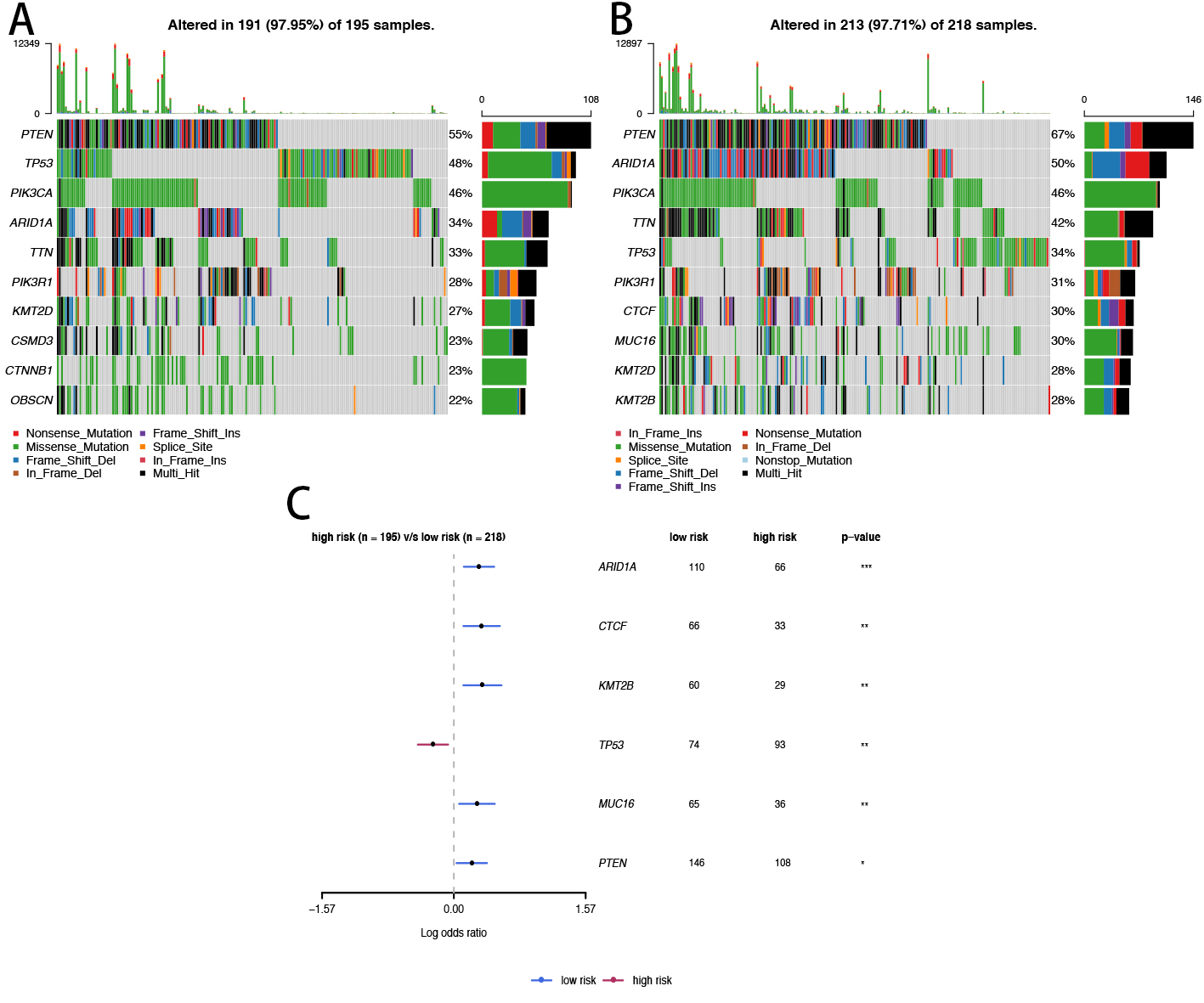

Supplement: Supplementary file 7 — Additional file 7: Figure S7. Mutational landscape of UCEC tumor samples in high- and low-risk groups. (A) Waterfall plot representing the mutational landscape of the top 10 most frequently mutated genes in high-risk. (B) Landscape of the top 10 most frequently mutated genes in high-risk and low-risk group. (C) Forest plot illustrating the genes that exhibit significant differences in mutational rate between high- and low-risk UCEC samples groups with the threshold of p < 0.05. [file 12935_2021_2038_MOESM7_ESM.jpg]

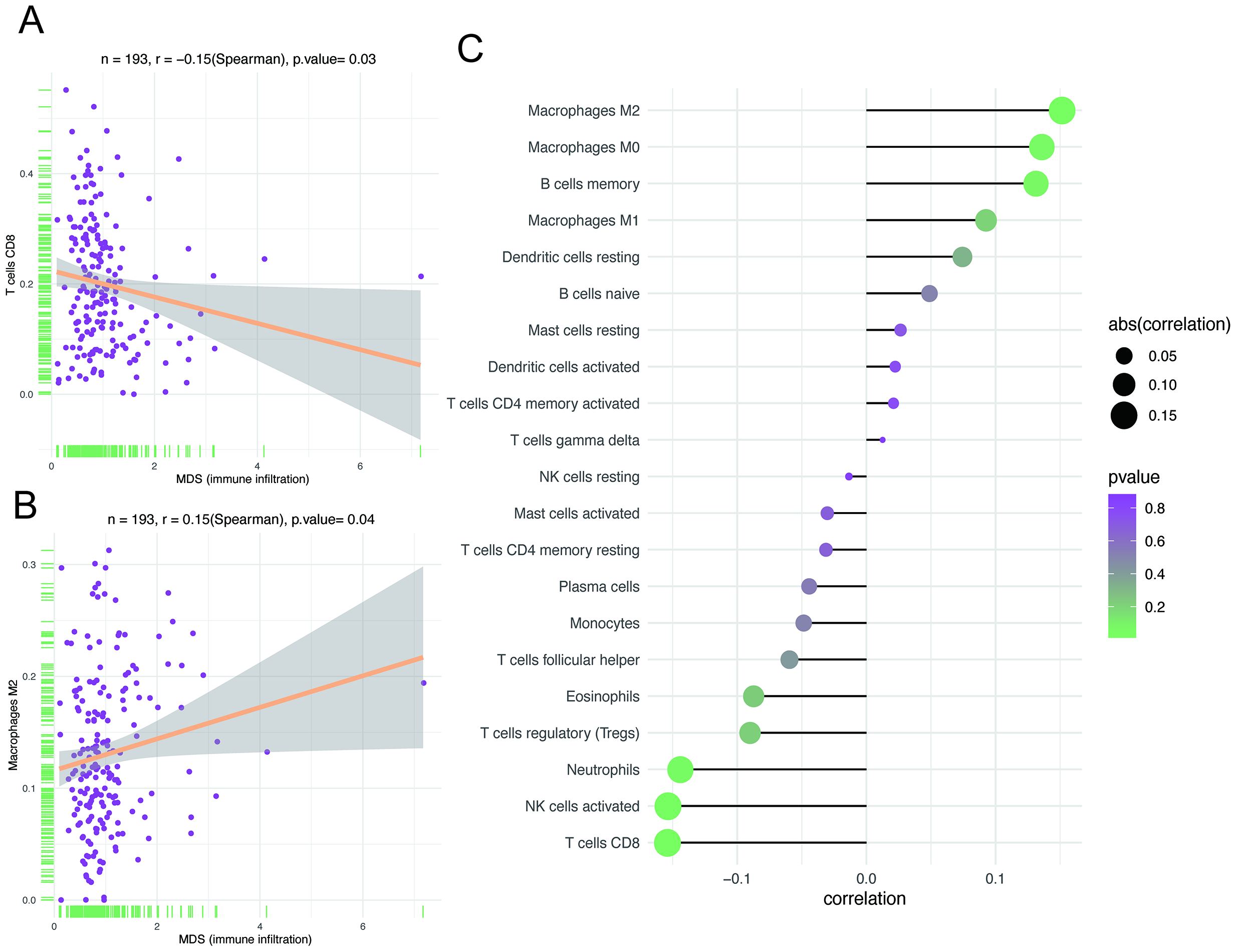

Supplement: Supplementary file 8 — Additional file 8: Figure S8. Correlation between MDS and immune cell infiltration. (A) The association between MDS and macrophage M2. (B) The association between MDS and T cells CD8. (C) The association between MDS and each type of immune cell. [file 12935_2021_2038_MOESM8_ESM.tif]

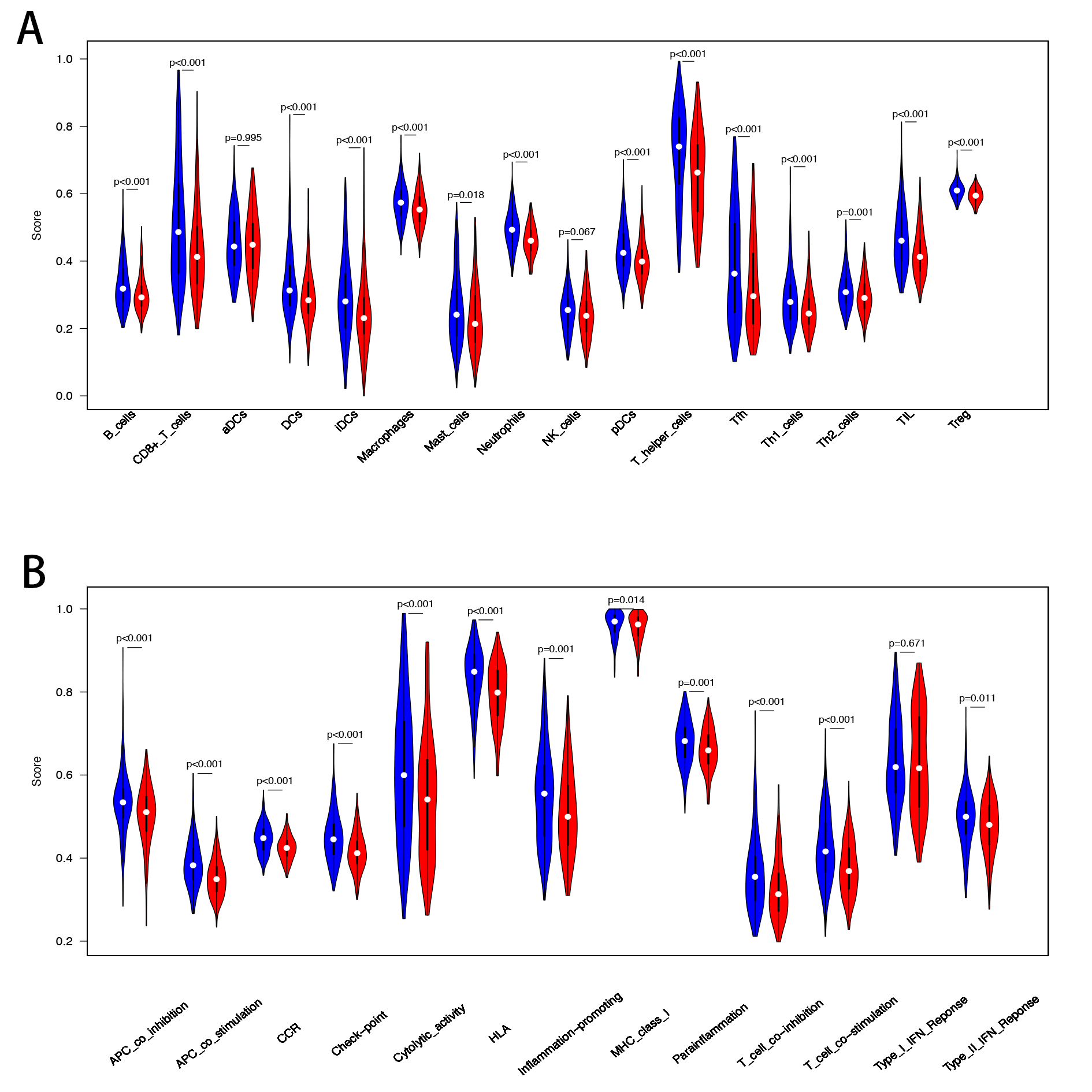

Supplement: Supplementary file 9 — Additional file 9: Figure S9. Comparison of the ssGSEA scores between different risk groups in the TCGA cohort. (A) The scores of 16 immune cells. (B) The scores of 13 immune-related functions. Red means high-risk group. Blue means low-risk group. [file 12935_2021_2038_MOESM9_ESM.jpg]

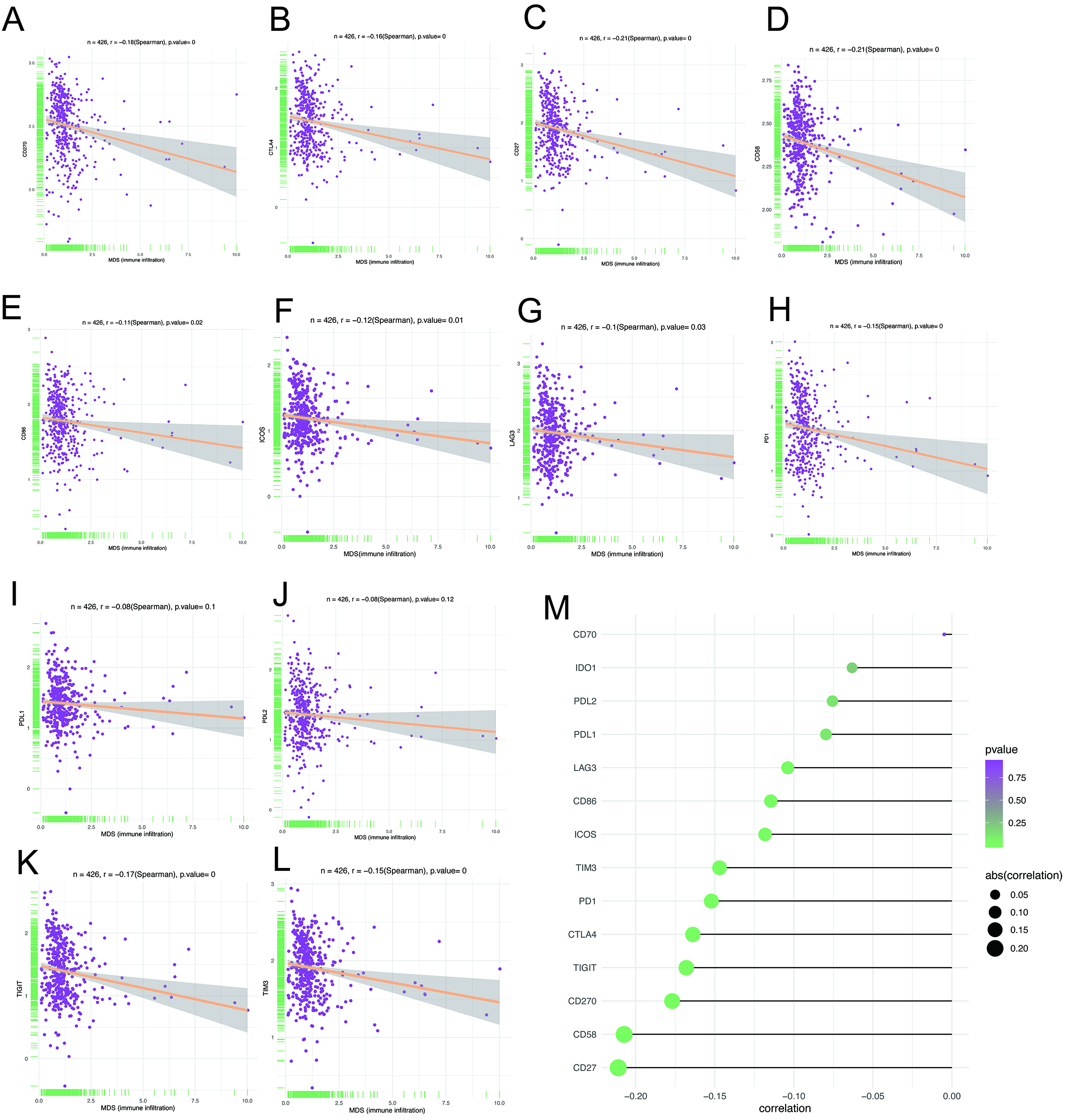

Supplement: Supplementary file 10 — Additional file 10: Figure S10. Correlation between MDS and the expression of checkpoints. (A–L) The association between MDS and each checkpoint. (M) The landscape of the association between MDS and some immune checkpoint regulators. [file 12935_2021_2038_MOESM10_ESM.tif]

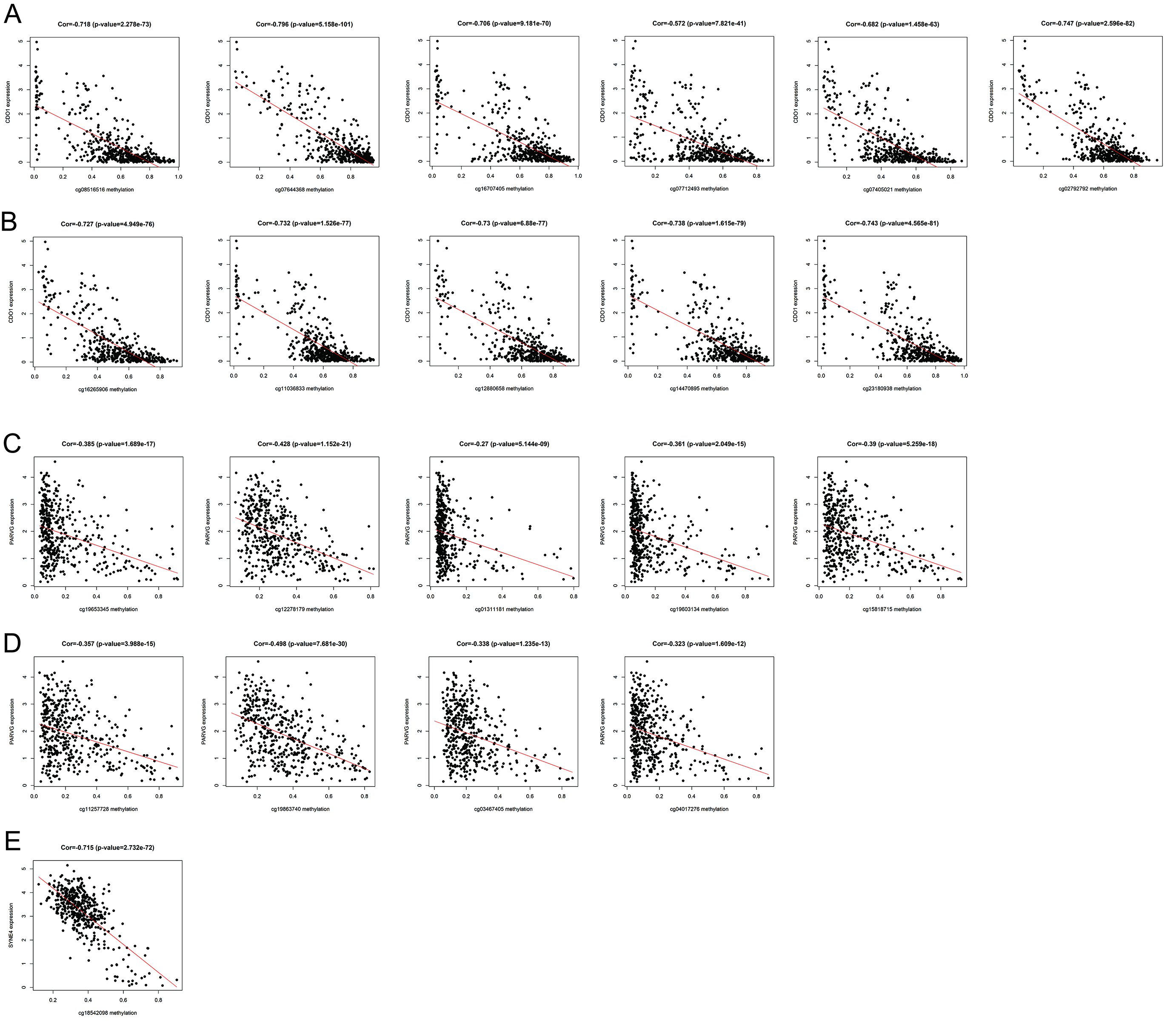

Supplement: Supplementary file 11 — Additional file 11: Figure S11. Correlation between methylation sites and the expression of 3 DMDGs. (A, B) The methylation level of 11 methylation sites is negatively correlated with the expression level of CDO1. (C, D) methylation level of 9 methylation sites is negatively correlated with the expression level of PARVG. (E) The level of methylation at a methylation site is negatively correlated with the expression level of SYNE4. [file 12935_2021_2038_MOESM11_ESM.tif]
