# Supplementary material for: Identification of methylation-driven genes prognosis signature and immune microenvironment in uterus corpus endometrial cancer
Source: Cancer Cell Int. 2021 Jul 10;21:365. doi: 10.1186/s12935-021-02038-z (PMC8272318; doi:10.1186/s12935-021-02038-z)
Supplement: Supplementary file 12 — Additional file 12: Table S1. Clinicopathologic characteristics of patients with TCGA-UCEC. [file 12935_2021_2038_MOESM12_ESM.docx]

**Table S1. Clinicopathologic characteristics of patients with TCGA-UCEC.**

| **Variables** | **Total (n=544) N(%)** |
| --- | --- |
| **Age** |  |
| ＜=60 | 208(38.2) |
| ＞60 | 336(61.8) |
| **Tumor type** |  |
| Endometrial cancer | 408(75.0) |
| Mixed and serous | 136(25.0) |
| **Grade** |  |
| G1 & G2 | 97(17.8) |
| G3 & G4 | 447(82.2) |
| **Stage** |  |
| Stage I & Stage II | 391(71.9) |
| Stage III Stage IV | 153(28.1) |
