# Supplementary material for: Identification of methylation-driven genes prognosis signature and immune microenvironment in uterus corpus endometrial cancer
Source: Cancer Cell Int. 2021 Jul 10;21:365. doi: 10.1186/s12935-021-02038-z (PMC8272318; doi:10.1186/s12935-021-02038-z)
Supplement: Supplementary file 13 — Additional file 13: Table S2. Primers’ information. [file 12935_2021_2038_MOESM13_ESM.docx]

**Table S2: Primers’ information.**

| **Gene** | **Forward primer** | **Reverse primer** |
| --- | --- | --- |
| CDO1 | 5' ATGGAACAGACCGAAGTGCTG 3' | 5' CTTGGCGTACATTGCCCAC 3' |
| SYNE4 | 5' CCTAGAGAGGCGGACATTGTT 3' | 5' AAGAGGGTGTTGACCATCTCG 3' |
| PARVG | 5' AGGTCATCACTATCGAGAGCAC 3' | 5' CCTCTTTCACTGCGTTCACTTTC 3' |
| GAPDH | 5' ACCACAGTCCATGCCATCAC 3' | 5' TCTAGACGGCAGG TCAGGTC 3' |
